# Lipopolysaccharides induced inflammatory responses and electrophysiological dysfunctions in human-induced pluripotent stem cell derived cardiomyocytes

**DOI:** 10.1038/s41598-017-03147-4

**Published:** 2017-06-07

**Authors:** Gökhan Yücel, Zhihan Zhao, Ibrahim El-Battrawy, Huan Lan, Siegfried Lang, Xin Li, Fanis Buljubasic, Wolfram-Hubertus Zimmermann, Lukas Cyganek, Jochen Utikal, Ursula Ravens, Thomas Wieland, Martin Borggrefe, Xiao-Bo Zhou, Ibrahim Akin

**Affiliations:** 1 First Department of Medicine, Faculty of Medicine, University Medical Centre Mannheim (UMM), University of Heidelberg, Mannheim, Germany; 2 DZHK (German Center for Cardiovascular Research), Partner Sites, Heidelberg-Mannheim Göttingen, Germany; 3 Institute of Pharmacology and Toxicology, University of Göttingen, Göttingen, Germany; 4 Skin Cancer Unit, German Cancer Research Center (DKFZ), Heidelberg and Department of Dermatology, Venereology and Allergology, University Medical Center Mannheim, University of Heidelberg, Mannheim, Germany; 5 Institute of Experimental and Clinical Pharmacology and Toxicology, Medical Faculty Mannheim, University of Heidelberg, Mannheim, Germany; 6 Key Laboratory of Medical Electrophysiology of Ministry of Education, Institute of Cardiovascular Research, Southwest Medical University, Luzhou, Sichuan China; 7 Stem Cell Unit, Heart Research Center Göttingen, Göttingen, Germany; 8 Institute of Experimental Cardiovascular Medicine, University Heart Centre Freiburg∙Bad Krozingen, Freiburg, Germany

## Abstract

Severe infections like sepsis lead frequently to cardiomyopathy. The mechanisms are unclear and an optimal therapy for septic cardiomyopathy still lacks. The aim of this study is to establish an endotoxin-induced inflammatory model using human induced pluripotent stem cell (hiPSC) derived cardiomyocytes (hiPSC-CMs) for mechanistic and therapeutic studies. hiPSC-CMs were treated by lipopolysaccharide (LPS) in different concentrations for different times. ELISA, FACS, qPCR, and patch-clamp techniques were used for the study. TLR4 (Toll-like receptor 4) and its associated proteins, CD14, LBP (lipopolysaccharide binding protein), TIRAP (toll-interleukin 1 receptor domain containing adaptor protein), Ly96 (lymphocyte antigen 96) and nuclear factor kappa B as well as some pro-and anti-inflammatory factors are expressed in hiPSC-CMs. LPS-treatment for 6 hours increased the expression levels of pro-inflammatory and chemotactic cytokines (TNF-a, IL-1ß, IL-6, CCL2, CCL5, IL-8), whereas 48 hour-treatment elevated the expression of anti-inflammatory factors (IL-10 and IL-6). LPS led to cell injury resulting from exaggerated cell apoptosis and necrosis. Finally, LPS inhibited small conductance Ca^2+^-activated K^+^ channel currents, enhanced Na^+^/Ca^2+^-exchanger currents, prolonged action potential duration, suggesting cellular electrical dysfunctions. Our data demonstrate that hiPSC-CMs possess the functional reaction system involved in endotoxin-induced inflammation and can model some bacterium-induced inflammatory responses in cardiac myocytes.

## Introduction

A severe infection by Gram-negative bacteria, like sepsis, can lead to multi organ dysfunctions including heart failure and arrhythmias^1, 2^. Lipopolysaccharide (LPS), the major component of the outer membrane of Gram-negative bacteria, is known to be a key pathogenic stimulator for the dysfunctions. Under septic circumstances circulating LPS as a pathogen associated molecular pattern (PAMP) can stimulate the innate immune system, which mediates a local or systemic inflammatory response. LPS can also stimulate non-immune cells and initiate the inflammatory process. Exaggerated inflammatory responses are usually detrimental. The innate LPS-pattern recognition receptor, the Toll-like receptor 4 (TLR4) is widely expressed in the body including cardiomyocytes^3, 4^. Therefore, the innate inflammatory response can be evoked in cardiomyocytes by LPS irrespective of the involvement of immune cells. This could be a reason for the facts that cardiac dysfunctions were frequently observed in patients with sepsis and also in animals injected with LPS^5–7^. The underlying mechanisms, however, remain largely unclear.

Two hypotheses have been proposed to explain sepsis-induced cardiac dysfunctions, i.e., inadequate coronary blood flow and chemical mediators were supposed to be causative for the dysfunctions. The former was based on studies in animals^8, 9^, showing that coronary blood flow was reduced by infusion of endotoxin. This theory, however, was refuted after other reports showing a marked coronary vasodilation and even higher coronary flow in patients with sepsis^10, 11^. The later was supported by increasing evidences. Administration of endotoxin depressed cardiac function^12, 13^. Cardiomyocytes exposed to the serum of patients with sepsis displayed impaired cell shortening and the causative factor has been proved to be the tumor necrosis factor alpha (TNF-a)^14–16^. Later, interleukin-1ß, nitric oxide, and reactive oxygen species (ROS), have been implicated in pathogenesis^17–19^. Despite the improvements in our current understanding of cardiac dysfunction in sepsis, the exact mechanisms have till now not been clarified. Therefore optimal therapy for septic cardiomyopathy still lacks and the mortality of patients with septic cardiomyopathy stays still high^19^.

To explore the signaling involved in the pathogenesis of septic cardiomyopathy, animals or animal cells have been frequently employed for different studies. Due to the differences between man and animals, animals or animal cells are not ideal for mimicking the human cardiac diseases. Because of limited availability and difficulty in long-time culture of human cardiomyocytes, some human cell lines, e.g., HEK293 and HeLa cells have been used for some studies investigating some inflammation-related signaling^20–22^. These non-cardiac cells are not ideal for functional studies because they cannot generate action potential and contraction. Since the successful reprogramming of adult somatic cells to induced pluripotent stem (iPS) cells and generation of functional cardiomyocytes from human iPS cells (hiPSC-CM)^23–26^, hiPSC-CMs have been demonstrated to have the electrophysiological and pharmacological properties including action potentials and responses to antiarrhythmic drugs which are similar to those of native cardiomyocytes^27, 28^. Therefore hiPSC-CMs can be a good alternative for modeling cardiac diseases. This has been confirmed by emerging evidences showing that the hiPSC-CMs derived from patients with genetic heart diseases recapitulated the phenotype of the disease^29–31^. Recently, it has been reported that hiPSC-CMs can model the coxsackievirus B3-induced myocarditis^32^. Here, we report that hiPSC-CMs can recapitulate LPS-induced inflammatory responses with electrophysiological dysfunctions and can serve as an *in vitro* model for mechanistic and drug-screening studies for some bacterium-induced inflammatory cardiomyopathy.

## Results

### LPS induced inflammatory responses in hiPSC-CMs

**Figure 1:**
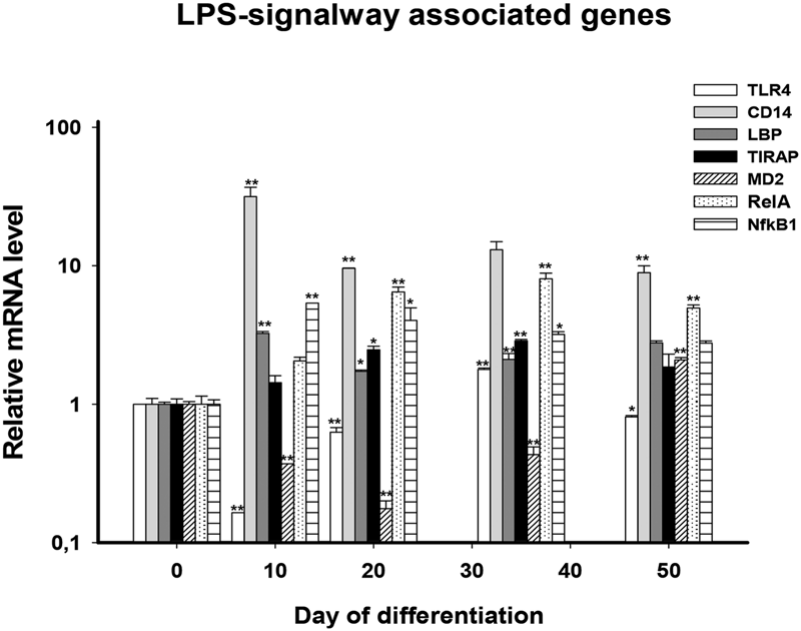
Expression of TLR4-associated signaling genes. Shown are the relative mRNA expression levels of LPS-signaling associated genes at different times of cardiac differentiation process (day 10 to day 50). The hiPSC-status at day 0 was used as relative control. *p < 0.05, **p < 0.01, ***p < 0.001 vs. day 0.

To check whether LPS-challenge can induce inflammatory responses in hiPSC-CMs, we first investigated the mRNA expression of LPS-receptor, the Toll-like receptor 4 (TLR4), and its associated signaling proteins in cells during the cardiac differentiation process. The qPCR-analysis showed that TLR4 and its associated proteins, CD14, LBP (lipopolysaccharide binding protein), TIRAP (toll-interleukin 1 receptor domain containing adaptor protein) and Ly96 (lymphocyte antigen 96, also known as MD2) are expressed in hiPSC-CMs. In addition, the expression of the gene RelA and NF-κB1, two subunits of nuclear factor-κB (NF-κB), an important downstream signaling factor of TLR4, was also detected (Fig. 1). Notably, on day 35 and 50 after differentiation, during which cells were used for the following studies, the mRNA expression levels of the receptor-associated proteins except MD2 on day 35 and TLR4 on day 50 are higher in hiPSC-CMs than that in hiPS cells.

**Figure 2:**
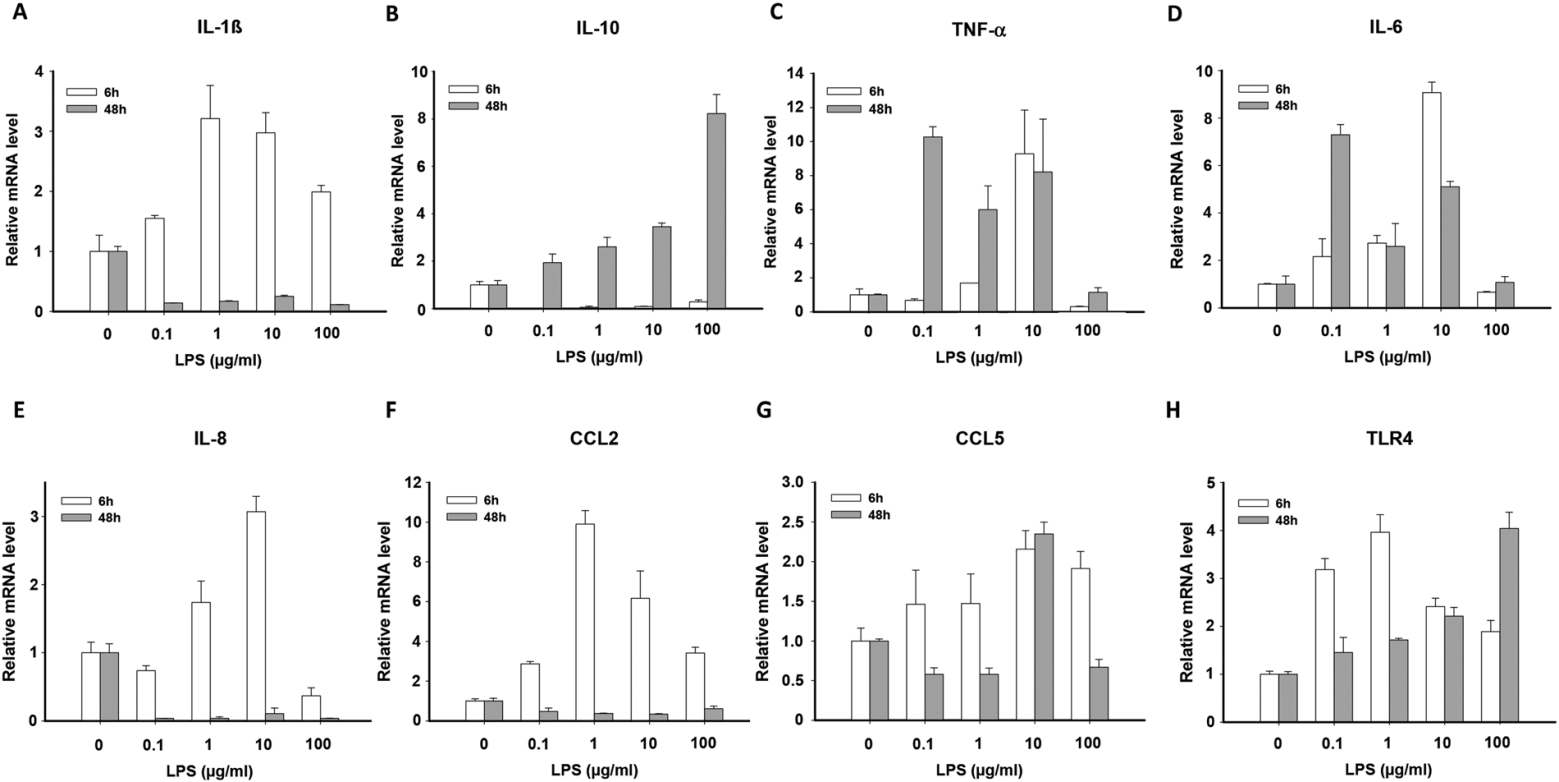
LPS-induced changes in the expression of inflammatory cytokines. Shown are the relative mRNA expression levels of different cytokines after LPS treatment in increasing concentrations for 6 h (white bar) and 48 h (gray bar). 6 hours treatment increased mRNA expression of pro-inflammatory factors IL1 (**A**), IL8 (**E**), CCL2 (**F**), and CCL5 (**G**), while prolonged treatment (48 h) enhanced expression of anti-inflammatory genes of IL10 (**B**). TNFα (**C**) and IL6 (**D**) were increased by LPS-treatment for both 6 h and 48 h. Main LPS-receptor TLR4 (**H**) was time-independently raised by LPS-treatment. *p*-values (One-way ANOVA) for 6 h- and 48 h-treatment (6 h/48 h): CCL2: 0.008/0.0156; CCL5: 0.0855/0.001; IL-1b: 0.0038/ < 0.0001; IL-6: <0.0001/0.0002; IL-8: 0.0008/0.005; TLR4: 0.0021/0.0011; TNF-α: 0.0012/0.0171; IL-10: 0.0010/0.0072.

Then we analyzed the mRNA expression of inflammatory factors in hiPSC-CMs treated by different concentrations of LPS for different incubation times. The qPCR-analysis was performed at 6 and 48 hours after LPS-challenge to check whether there are differences of pro- and anti-inflammatory response in acute and prolonged LPS-exposure. The results displayed that the mRNA expression levels of interleukin-1ß (IL-1ß) was increased by LPS treatment for 6 hours (Fig. 2A), reflecting its roles in the pro-inflammatory responses, whereas interleukin-10 (IL-10) was increased by LPS only at 48 hour-treatment (Fig. 2B), consistent with its anti-inflammatory effects. TNF-α, an important mediator of some acute and chronic inflammation, and interleukin-6 (IL-6), a well-known cytokine with pro- and anti-inflammatory properties during acute phase response, was enhanced by LPS-treatment at both 6 and 48 hours (Fig. 2C,D). In addition, the mRNA expression of some chemotactic cytokines, e.g., interleukin-8 (IL-8), CCL2 and CCL5, also increased at 6 hours after LPS-challenge (Fig. 2E–G). Furthermore, TLR4 mRNA expression levels were elevated by LPS irrespective of treating time (Fig. 2H).

To check whether the results on mRNA level could be confirmed on protein level we exemplarily applied an ELISA analysis for human IL-6. We found that the detectable IL-6 protein in the supernatants of hiPSC-CMs was also increased after LPS-treatment for 6 h and 48 h (Figure S1A). Then we used fluorescence-activated cell sorting (FACS) to check the receptors of IL-6 in LPS-treated hiPSC-CMs. We could demonstrate that CD 126, a major receptor of IL-6, was undetectable in hiPSC-CMs either with or without LPS-challenging (Figure S1B, p3 gate), whereas cardiac troponin T (TNNT) was detected in 50–90% of the cells (Figure S1B, p2 gate). These data suggest that CD126 does not exist in hiPSC-CMs. Further, we analyzed soluble CD130 (sCD130), a well-known IL-6 signal transducer^33, 34^. We observed a significant increase of sCD130 in hiPSC-CMs supernatants after prolonged LPS-treatment (48 h) with higher concentrations (Figure S1C).

For optical verifying of our results we further established an immunohistochemistry protocol. First we stained our hiPSC-CMs monolayer with cardiac TNNT. Nuclear staining was performed with DAPI. We could show striated cardiac muscle cells (Figure S2A,B). In the next step we supplemented a titin antibody to the first protocol, where we could detect the structural proximity of both proteins in cardiac muscle cells (Figure S2C). Finally we applied 10 µg/ml LPS on hiPSC-CMs monolayer and stained after 6 h treatment with a NF-κB antibody, which resulted in nuclear-near fluorescence signals (Figure S2D).

### LPS induced cell injury

**Figure 3:**
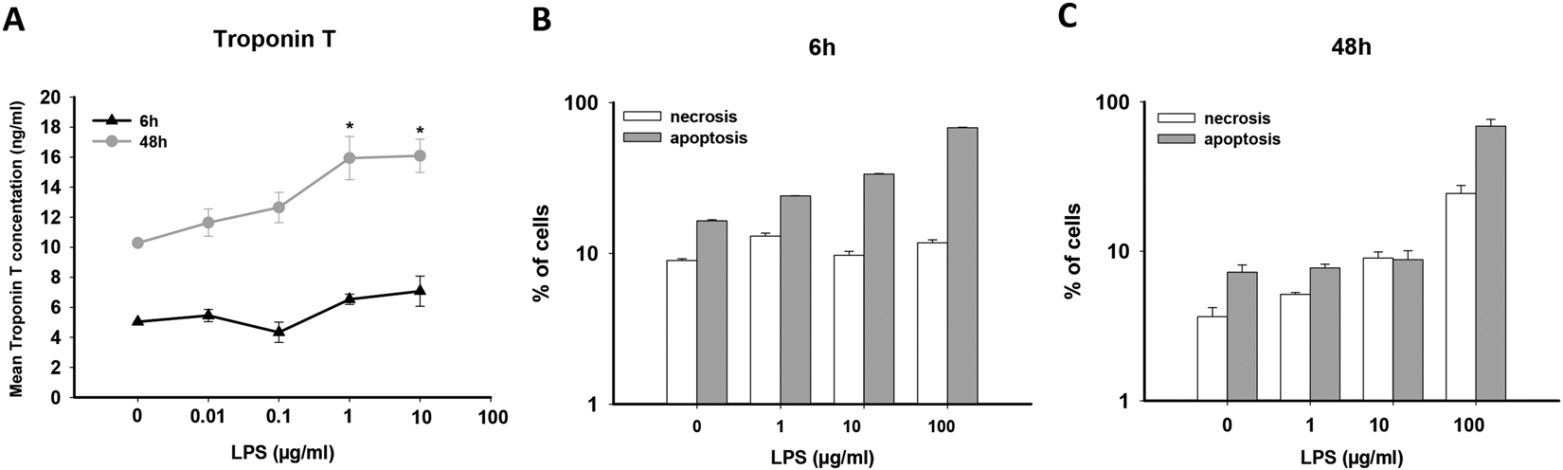
LPS induced cell impairment. (**A**) ELISA analysis showing that 48 h-but not 6 h-treatment with LPS increased concentrations of human Troponin T (TNNT) in **s**upernatants of LPS-treated hiPSC-CMs. *p*-values (test for linear trend): 6 h > 0.05, 48 h < 0.05. (**B**,**C**) FACS analysis for necrosis (7AAD positive) and apoptosis (Annexin-V positive) ratio of hiPS-CMs treated by LPS in different concentrations for 6 and 48 hours. 6 h-treatment showed a dose dependent increase of apoptotic cells ratio, but no influence on necrotic ratio (**B**). 48 h-treatment showed a dose dependent increase of necrotic cells ratio (**C**). The increase in apoptotic ratio was observed only when LPS concentration was raised to 100 µg/ml (*p* < 0.001 vs. 0 µg/ml). Test for linear trend: 6 h, necrosis, *p* > 0.05, apoptosis, *p* < 0.001; 48 h, necrosis, *p = *0.003.

Cell injury happens frequently in inflammatory responses. Troponin measurement for sepsis-induced cardiac damage was previously described^35^. Therefore we assessed the cell injury in hiPSC-CMs using different techniques. First we used ELISA to measure the concentration of TNNT in culture medium with the same amount of cells treated either with vehicle or different concentrations of LPS for 6 and 48 hours. We found that the TNNT level in the culture medium was not significantly changed by LPS-treatment for 6 hours but increased by LPS-treatment for 48 hours in a dose-dependent manner (p < 0.05, one-way ANOVA, Fig. 3A).

Then we examined apoptosis- and necrosis- rate of hiPSC-CMs challenged by LPS. For this aim, FACS with a Annexin-V-APC/7-AAD conjugated antibody was employed to analyze the rate of apoptotic and necrotic cells treated with 1, 10 and 100 µg/ml of LPS for 6 or 48 hours (Fig. 3B,C). In 6 hour-treatment, the rate of apoptosis but not necrosis was enhanced by LPS with increasing concentrations (Fig. 3B). In 48 hour-treatment, the dose-dependent enhancement of necrosis was clearly displayed, whereas the apoptosis rate was not elevated until 100 µg/ml LPS (Fig. 3C).

### LPS changed expression level of ion channels

**Figure 4:**
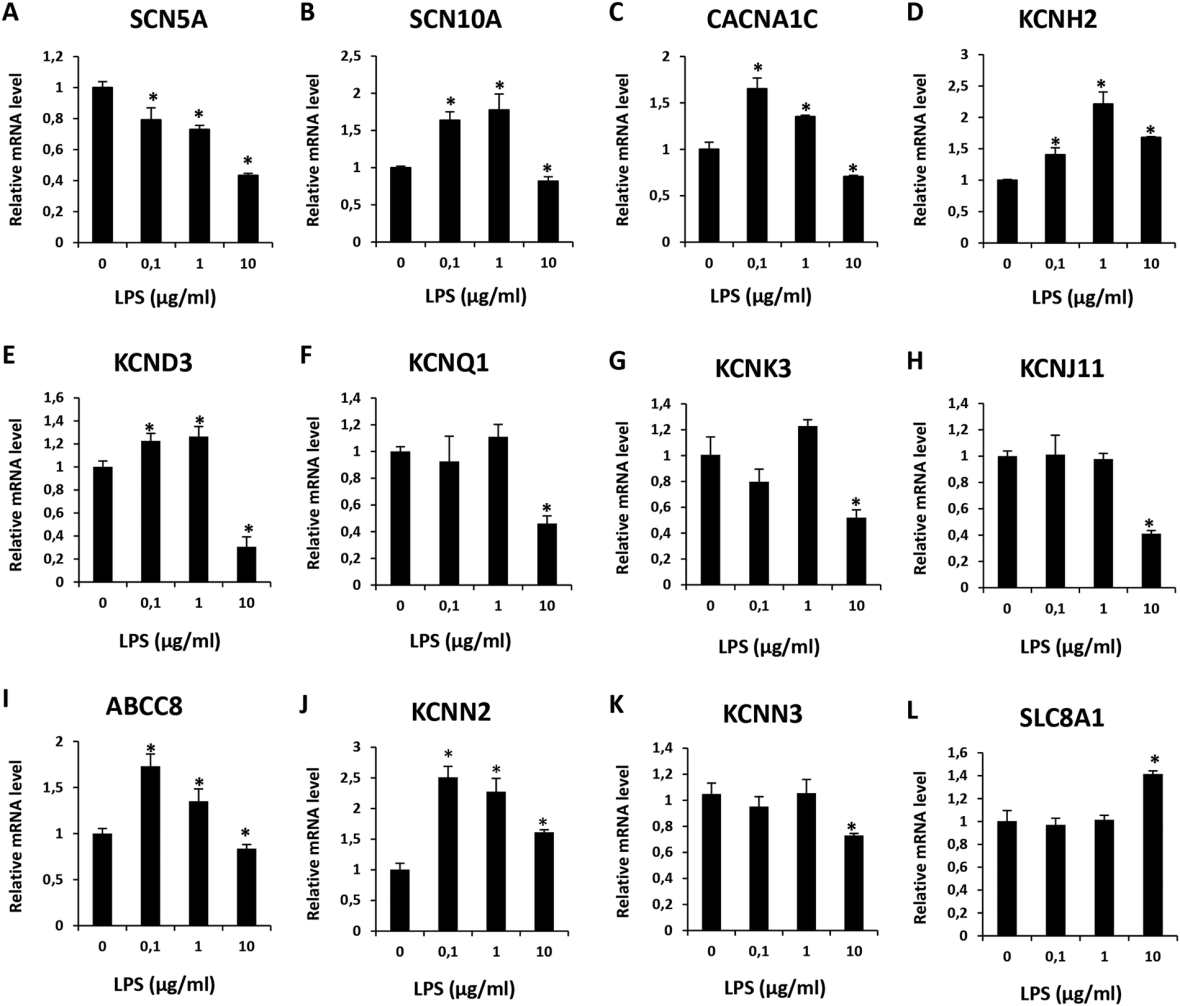
LPS-induced changes in mRNA expression of ion channels. Relative mRNA levels of different ion channels were analyzed by qPCR in hiPSC-CMs after treatment with LPS in different concentrations for 48 h, Values given are mean ± SEM. *p < 0.05 vs control (0 µg/ml).

The clinic fact that patients with severe infections have frequently heart dysfunctions including arrhythmias led us to check whether ion channel expression levels are also changed in hiPSC-CMs treated by LPS. To this end, hiPSC-CMs were treated for 48 hours by 3 concentrations of LPS (0.1, 1, and 10 µg/ml). Expression levels of mRNA of ion channels were analyzed by qPCR. LPS of 0.1 and 1 µg/ml reduced the expression level of SCN5A (Na^+^ channel, Nav1.5, Fig. 4A) but increased the expression of SCN10A (Na^+^ channel, Nav1.8, Fig. 4B), CACNA1C (L-type Ca^2+^ channel, Fig. 4C), KCNH2 (I_Kr_, Kv11.1, Fig. 4D), KCND3 (I_to_, Kv4.3, Fig. 4E), ABCC8 (K_ATP_, beta-subunit SUR1, Fig. 4I), KCNN2 (SK2, Fig. 4J). No significant changes in expression of KCNQ1 (I_Ks_, Kv7.1, Fig. 4F), KCNK3 (TASK-1, Fig. 4G), KCNJ11 (K_ATP_, alpha-subunit, Fig. 4H), KCNN3 (SK3, Fig. 4K), and SLC8A1 (NCX1, Fig. 4L) were detected. Remarkably, 10 µg/ml LPS reduced consistently the channel expression either significantly or by tendency except for KCNH2 and SLC8A1, probably due to cell injury by high concentration of LPS. Therefore, only the concentrations of 0.1 and 1 µg/ml were used for the following studies.

### LPS prolonged action potential duration (APD)

**Figure 5:**
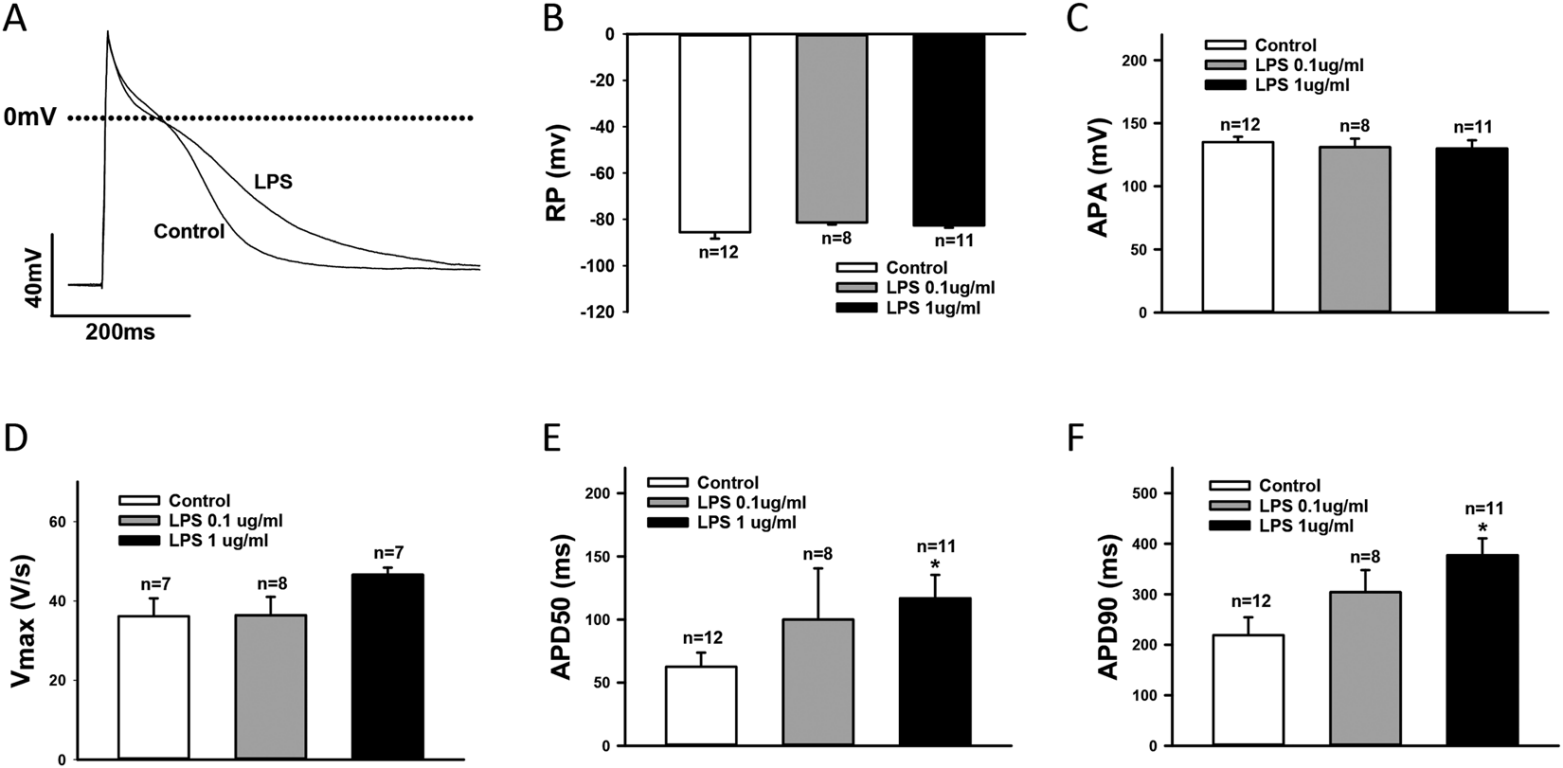
LPS-treatment prolonged APD. (**A**) Representative traces of action potentials (AP) in control and LPS-treated hiPSC-CMs. (**B**) Mean values of resting potentials (RP). (**C**) Mean values of action potential amplitude (APA). (**D**) Mean values of maximal upstroke velocity of AP (Vmax). (**E**) Mean values of APD at 50% repolarization (APD50). (**F**) Mean values of APD at 90% repolarization (APD90).Values given are mean ± SEM. n, number of cells. *p < 0.05.

To investigate the effects of LPS on the electrophysiological properties of cardiomyocytes, hiPSC-CMs were treated by 0.1 and 1 µg/ml LPS for 48 hours and then the membrane potentials and currents were measured. LPS-treatment did not change the RP (resting potential) and APA (amplitude of action potential), but enhanced Vmax (maximal velocity of depolarization) and prolonged both APD50 (repolarization at 50%) and APD90 (repolarization at 90%) significantly (Fig. 5). Vmax was enhanced from 36. ± 1 4.5 V/s to 46.6 ± 1.8 V/s (*p* = 0.054). APD50 was prolonged from 62.6 ± 11.2 ms to 116.8 ± 18.6 ms (*p* < 0.05) and APD90 from 219.1 ± 35.4 ms to 377.4 ± 33.5 (*p* < 0.05).

### Effects of LPS on sodium channel currents

**Figure 6:**
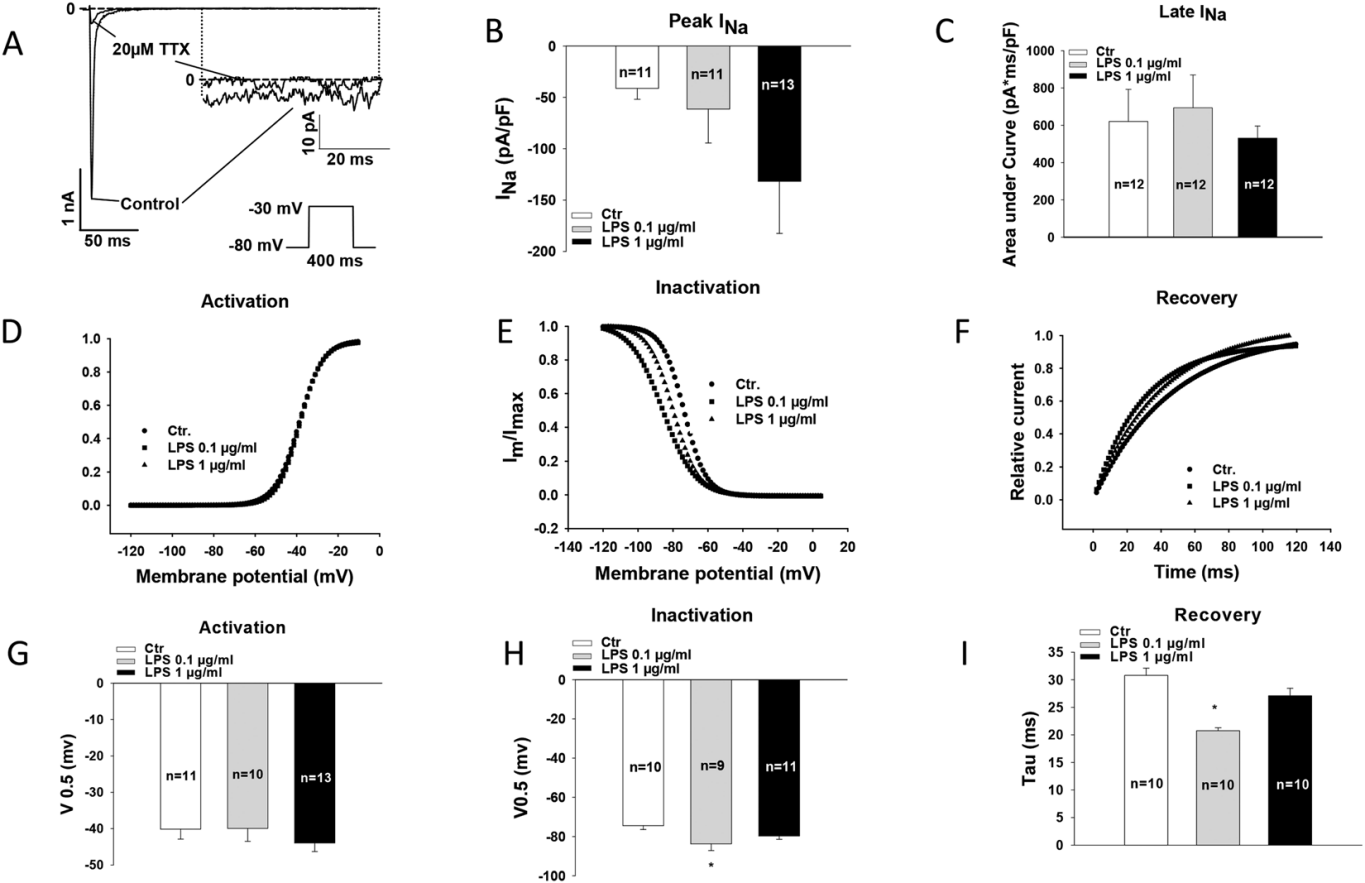
Effects of LPS on I_Na_. (**A**) Representative traces of I_Na_ at −30 mV in absence and presence of 20 µM TTX. (**B**) Averaged peak I_Na_ at −30 mV in control and LPS-treated hiPSC-CMs. (**C**) Averaged values of late I_Na_ at −30 mV. (**D**) Representative activation curves of peak I_Na_. (**E**) Representative inactivation curves of peak I_Na_. (**F**) Representative curves of recovery from inactivation of peak I_Na_. (**G**) Mean values of voltages at 50% of steady-state activation of peak I_Na_ (V0.5). (**H**) Mean values of voltages at 50% of steady-state inactivation of peak I_Na_. (**I**) Mean values of time constants (Tau) of recovery from inactivation of peak I_Na_. Values given are mean ± SEM. n, number of cells. *p < 0.05.

To assess the ion channel currents that may contribute to the observed changes of APs by LPS, sodium channel currents (I_Na_) were checked in LPS-treated hiPSC-CMs (Fig. 6). LPS (1 µg/ml) increased the peak I_Na_ but reduced the late I_Na_ (Fig. 6B,C). Although the changes did not reach statistical significance, the test for linear trend showed a tendency of significance (*p* = 0.08). The peak I_Na_ was changed by 1 µg/ml LPS from 41.2 ± 10.7 to 131.7 ± 50.7 pA/pF (p > 0.05,) and the late I_Na_ from 620.6 ± 172.2 to 531.1 ± 64.8 pA*mV/pF (p > 0.05), respectively. No changes in the activation of I_Na_ were detected (Fig. 6D and G). A shift to more negative potential of the inactivation curve was observed in cells treated by 0.1 µg/ml LPS (Fig. 6E and H), i.e, V0.5 (the half maximal voltage of inactivation) was shifted from −74.4 ± 1.9 to −83.7 ± 3.4 mV (p < 0.05). In addition, the recovery from inactivation of I_Na_ was speeded up by 0.1 µg/ml LPS (Tau was reduced from 31.0 ± 1.3 to 20.8 ± 0.5 ms, p < 0.05, Fig. 6F and I).

### LPS reduced small conductance calcium-activated K^+^ channel currents (I_SK1–3_)

**Figure 7:**
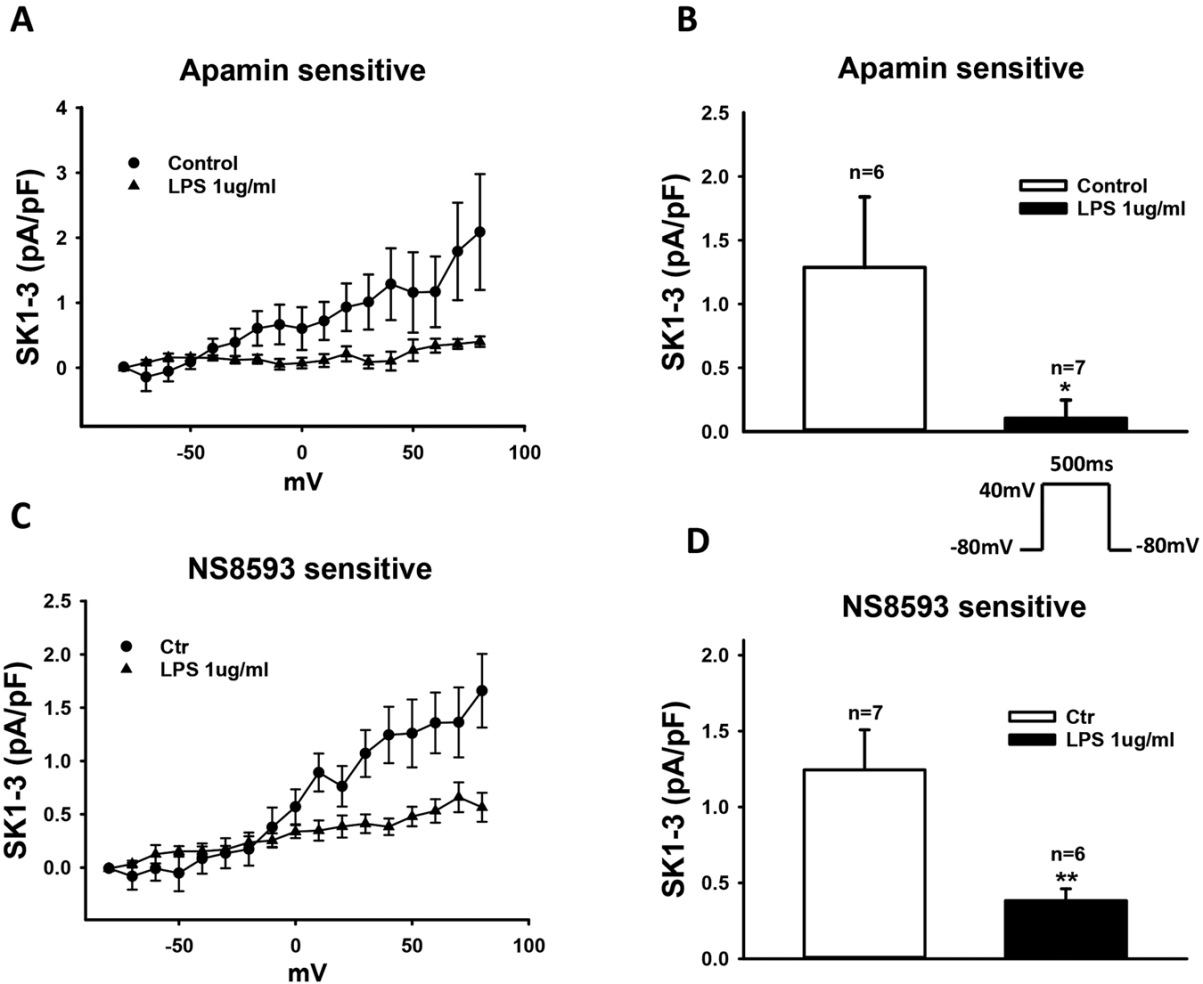
LPS-treatment attenuated SK1-3 channel currents (I_SK1-3_). Membrane currents were recorded in absence and presence of 100 nM apamin (**A** and **B**) or 10 µM NS8593 (**C** and **D**), blockers of SK1-3 channels. The blocker-sensitive currents were analyzed as I_SK1-3._ (**A**) Current-voltage (I–V) relationship curves of apamin-sensitive currents recorded from −80 to +80 mV with the holding potential of −80 mV. (**B**) Mean values of apamin-sensitive currents at +40 mV. (**C**) I–V curves of NS8593-sensitive currents. (**D**) Mean values of NS8593-sensitive currents at +40 mV. Values given are mean ± SEM. n, number of cells. *p < 0.05, **p < 0.01.

The detected effects of LPS on I_Na_ cannot explain the APD-prolongation. Therefore we further assessed the ion channel currents possible for prolonging APD. We found that I_SK1–3_ was significantly reduced by LPS-treatment (Fig. 7 and S3,S4). To separate I_SK1–3_ from other ion channel currents, two SK1–3 channel blockers, apamin (100 nM) and NS8593 (10 µM), were used. The blocker-sensitive currents were analyzed as I_SK1-3_. In the presence of 1 µg/ml LPS, apamin- and NS8593-sensitive currents (at 40 mV) were reduced from 1.3 ± 0.6 pA/pF to 0.1 ± 0.1 pA/pF (p < 0.05) and from 1.2 ± 0.3 pA/pF to 0.4 ± 0.1 pA/pF (p < 0.05), respectively.

### LPS enhanced Na/Ca-exchanger currents (I_NCX_)

**Figure 8:**
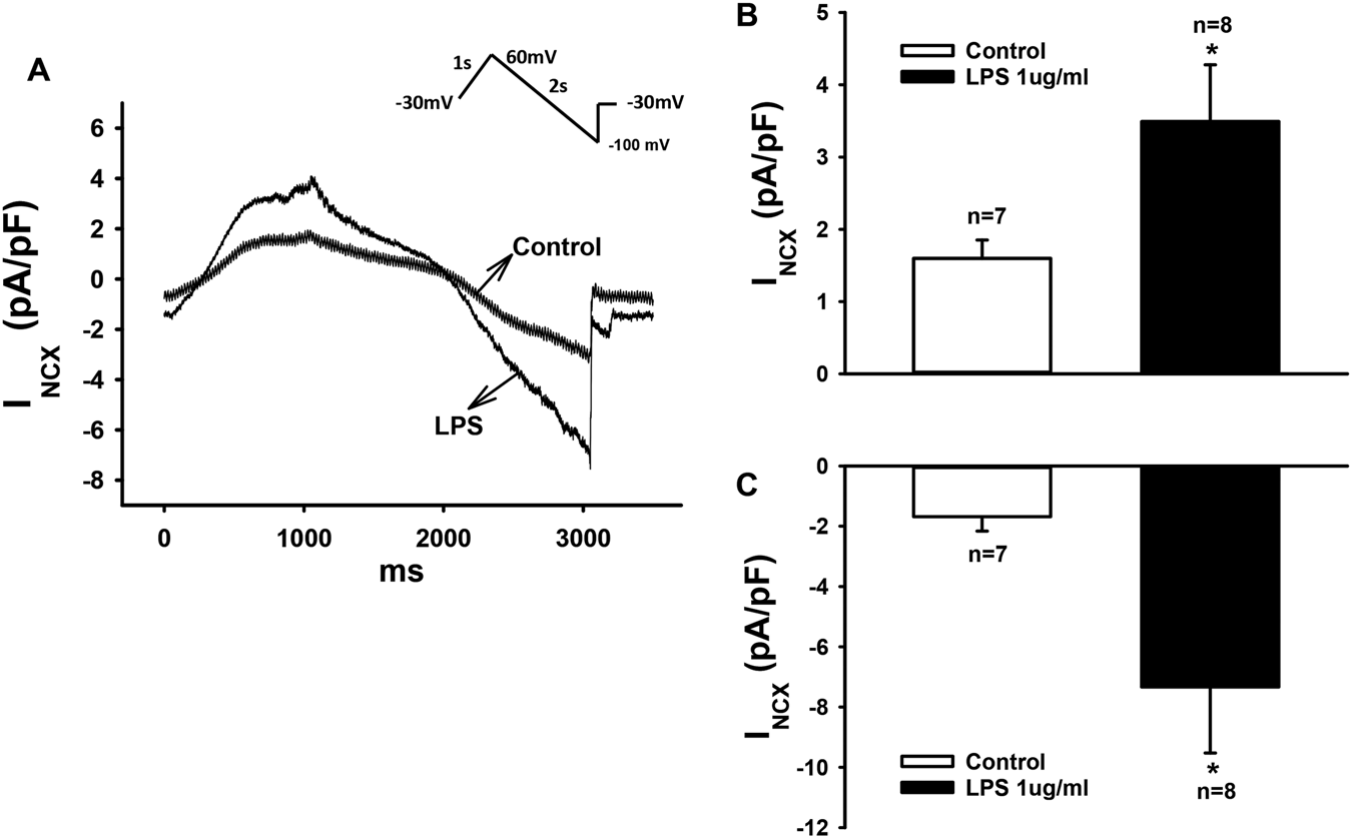
LPS-treatment enhanced Na/Ca exchanger currents (I_NCX_). I_NCX_ was evoked by ramp pulses (100 mV/s) at 0.5 Hz from −30 to +60, then to −100 mV with the holding potential of −30 mV. NiCl_2_ (5 mM) was used to isolate I_NCX_ from other currents. (**A**) Representative traces of Ni^2+^-sensitive currents in absence and presence of LPS (1 µg/ml for 48 hours). (**B**,**C**) Mean values of peak I_NCX_ at +60 and −100 mV. Values given are mean ± SEM. n, number of cells. *p < 0.05.

We also detected that I_NCX_ was significantly enhanced by LPS-treatment (Fig. 8). To separate the I_NCX_, 5 mM NiCl_2_, a NCX- blocker, was used. LPS of 1 µg/ml enhanced the Ni^2+^-sensitive currents (I_NCX_) at +60 mV and −100 mV from 1.6 ± 0.3 to 3.5 ± 0.8 pA/pF (p < 0.05) and from −1.7 ± 0.5 to −7.3 ± 2.2 pA/pF (p < 0.05), respectively.

### Effects of LPS on other ion channels that may influence APs

In addition to I_Na_, I_NCX_ and I_SK1-3_, many other ion channels are known to be important for APD. Therefore we checked the effects of LPS-treatment on different ion channels that may be involved in the APD-prolongation. LPS enhanced I_to_ (transient outward current, Figure S5A,B) but failed to change I_Ca-L_ (L-type Ca^2+^ channel currents, Figure S5C,D), I_Kr_ (rapidly activating delayed rectifier current, Figure S6A,B), I_Ks_ (slowly activating delayed rectifier current, Figure S6C,D), I_K-pH_ (pH-sensitive K^+^ channel currents, Figure S7A,C) and I_KATP_ (ATP-sensitive K^+^ channel current, Figure S7D–F).

### Effects of LPS on intracellular Ca^2+^ concentration ([Ca^2+^]_i_)

The prolonged APD and enhanced I_NCX_ may influence the intracellular Ca^2+^ level. Therefore we measured [Ca^2+^]_i_ by Ca^2+^ imaging with a Ca^2+^-sensitive fluorescent indicator fluo-3. LPS-treatment (1 µg/ml for 48 h) did not significantly influence systolic and diastolic Ca^2+^-levels (Figure S8).

## Discussion

To our knowledge, this is the first study to demonstrate that hiPSC-CMs possess the functional reaction system involved in endotoxin-induced inflammation and displayed inflammatory responses and ion channel dysfunctions when they were challenged by LPS, suggesting hiPSC-CMs as a successful model of inflammation in cardiomyocytes, which may be useful for future studies on some bacterium-induced inflammatory human cardiac diseases.

In our lab, hiPS-CMs have been successfully generated from a healthy donor with the majority of ventricular myocytes. We have demonstrated that the cells expressed cardiac specific proteins, like TNNT2, MYH6, NKX2.5, and ACTN2^36^. They exhibited cardiac striated structure (Figure S2A–C) and contracted either spontaneously or when stimulated. They possess most ion channels reported to exist in human cardiomyocytes and displayed electrophysiological properties similar to that of native cardiomyocytes. Therefore we tried to establish a hiPSC-CM-model mimicking LPS-induced inflammation in cardiac myocytes, which is still lacking.

To know whether hiPSC-CMs can model inflammation induced by LPS, we checked whether the correspondent innate signalings exist in hiPSC-CMs and whether the cells can respond to LPS-challenge. This study demonstrated by qPCR that hiPSC-CMs express the mRNA of the LPS-receptor, TLR4, and its associated signaling proteins, LBP, CD14, MD2, and TIRAP with higher levels at days 35 or 50 after start of differentiation than that at day 0 (Fig. 1). This is consistent with previous findings showing the expression of TLR4 in human cardiomyocytes^3, 4^. We also detected the mRNA expression of RelA and NFkB1, subunits of the transcription factor NF-kB, which is an important downstream factor of TLR4, being higher at day 50 after differentiation than at day 0.

Activation of NF- kB can induce production of different inflammatory factors. We found out that hiPSC-CMs, like native cardiomyocytes, did express pro-inflammatory and anti-inflammatory as well as chemotactic cytokines when stimulated by LPS in different concentrations for different incubation times (Fig. 2). The well-known pro-inflammatory factor, IL-1ß, and also the chemotactic cytokines, CCL2, CCL5 and IL-8 showed a dose-dependent increase of mRNA expression level after LPS-treatment for 6 hours, suggesting their contribution to the early pro-inflammatory and chemotactic reaction phase of inflammation. On the other hand, the expression of the well-known anti-inflammatory cytokine IL-10 was increased at 48 hour- but not 6 hour-treatment with LPS (Fig. 2B), which is indicative of the initiation of anti-inflammatory processes under prolonged inflammatory circumstances. The mRNA level of TNF-α and IL-6, the important downstream factors of NF- kB, was increased in both early (6 hours) and late (48 hours) LPS-exposure (Fig. 2C,D), which was consistent with their pro- and anti-inflammatory functions. TLR4 expression was also raised by LPS-challenge, consistent with a previous report^3^. Moreover, immunohistochemistry-analysis showed a nuclear-near signal for NF-kB-targeting antibody after LPS-treatment (Figure S2D), suggesting that the LPS-mediated signaling led to the activation and nuclear translocation of the transcription factor NF-kB.

To further analyze the LPS/NF-kB-signaling, we analyzed IL-6 and its receptors, which have been shown to be involved in some heart diseases^35^. We first checked the protein-level of IL-6 in hiPSC-CMs treated with LPS. The detectable IL-6 protein levels in hiPSC-CM supernatants were elevated by LPS-treatment for 6 and 48 hours (Figure S1A), which is consistent with the qPCR-data and shows the correlation of transcriptome and protein level in the inflammatory response. It is known that IL-6 initiates inflammatory responses through either the membrane-bound or soluble IL-6 receptors (CD126). The membrane-bound CD126 exists predominantly in hepatocytes, neutrophils, monocytes/macrophages, and some lymphocytes^37^. The circulating soluble form of CD126, which can be detected in various body fluids, is secreted by monocytes, hepatocytes, and endothelial cells^37^. Both membrane-bound and soluble CD126 are associated with the signal transducing receptor glycoprotein 130 (gp130, also known as CD130, IL6ST, IL6-beta). The membrane-bound CD126-mediated signaling (classical IL-6 signaling) can be initiated only in cells expressing CD126. In the soluble CD126-mediated signaling (IL-6 trans-signaling), cells do not have to express CD126, as CD130 is widely expressed in most cell types. Therefore, we checked the LPS-treated-hiPSC-CMs for the presence of CD126 and CD130. As we expected, FACS analysis showed no fluorescence signal detectable for CD126 irrespective of LPS-challenge (Figure S1B). However, soluble CD130 (sCD130) was detectable in the hiPSC-CM supernatants and the protein-level was raised by prolonged LPS-exposure (Figure S1C). This may imply that a local IL-6 trans-signaling, like in native cardiomyocytes, can take place in the cells. Taking together, it is quite convincing that in hiPSC-CMs LPS stimulated TLR4 and activated NF-kB signaling, which in turn elevated production of inflammatory factors. The inflammatory factors may trigger inflammatory responses in cells.

The inflammation may result in cell injury. Therefore we measured the cardiac TNNT in the cell culture supernatant from hiPSC-CMs treated by LPS (Fig. 3A). Acute (6 hours) LPS-exposure did not influence the TNNT-level, indicative of no manifest cell injury. When applied for longer time (48 hours), LPS increased the TNNT-level in a dose-dependent manner. This implies that either stronger or longer inflammatory responses may enhance the cell impairment.

The cell injury may result from cell apoptosis and necrosis. Our data demonstrated that short time (6 hours) LPS-treatment increased the apoptosis but not necrosis ratio in a concentration dependent manner, while long time (48 hours) treatment with LPS enhanced necrotic cells ratio with increasing concentrations (Fig. 3B,C). These data, together with the TNNT-analysis, suggest that in the early phase of inflammation the cell injury was slight and resulted mainly from apoptosis induction through LPS associated signal ways. Prolonged LPS exposure led to severe injury because of a higher proportion of necrotic cells.

It is well-known that some infections, especially some severe infections like sepsis, may cause cardiac dysfunctions including arrhythmias. In this study, hiPSC-CMs treated by LPS displayed APD-prolongation (Fig. 5). APD-prolongation may be arrhythmogenic, which may explain, at least partially, the tachyarrhythmias observed in inflammatory models of animal or in patients with sepsis^2, 38–40^.

To understand the reason for APD-prolongation, we assessed the changes in ion channel currents that may contribute to APD. We found that peak I_Na_ was enhanced but late I_Na_ was reduced by LPS (Fig. 6). Reduced late I_Na_ should shorten APD, instead of prolonging APD. Next, we observed that LPS increased I_to_ significantly (Figure S5A,B), which should shorten APD, too. Furthermore, no significant changes in I_Ca-L_ (Figure S5C,D), I_Kr_ (Figure S6A,B), I_Ks_ (Figure S6C,D), pH-sensitive K^+^ channel currents including acidosis-and alkaline-inhibited K^+^ channel currents (Figure S7A–C), and K_ATP_ (Figure S7D–F) channel currents were detected in LPS-treated cells. All of these data are not consistent with the APD-prolongation. However, we found that LPS reduced I_SK1-3_ and enhanced I_NCX_ (Figs 7 and 8), both of which can result in ADP-prolongation. Increased I_NCX_ can also lead to DAD and in turn to tachyarrhythmias. This may be a reason for arrhythmias in patients with sepsis. APD-prolongation and enhanced I_NCX_ may elevate intracellular Ca^2+^-concentration. But we did not observe significant changes of systolic and diastolic Ca^2+^-levels in cells treated by LPS (1 µg/ml for 48 h). This may suggest extra effects of LPS that counteracted the effects of APD-prolongation and I_NCX_-enhancement on intracellular Ca^2+^-concentration.

To investigate the reason for changes of ion channel currents, we analyzed the channel mRNA expression levels with the qPCR technique (Fig. 4). The mRNA levels of the studied channels were changed by LPS in a concentration-dependent manner. High concentration of LPS (10 µg/ml) consistently reduced expression of all the channels except NCX1 (Fig. 4), suggesting toxic effects and cell injury. Low concentrations (0.1 or 1 µg/ml) of LPS influenced differentially the ion channel expression. SCN5A was reduced but SCN10A was increased (Fig. 4A and B). Whether the former led to reduction in late I_Na_ (Fig. 6C) and the latter to the enhancement of peak I_Na_ (Fig. 6B) cannot be decided because the contributions of both channel types to I_Na_ were not assessed in this study. The expression of SK2 but not SK3 channel, which have been reported to be the important SK channel isoforms in human cardiomyocytes^41^, was increased (Fig. 4J). This is not consistent with the reduced I_SK1-3_ (Fig. 7). Whether the protein levels of SK2 and SK3 changed in LPS-treatment needs to be addressed. No changes of LSC8A1 (NCX1, Fig. 4L) expression were detected, this did not fit the enhanced I_NCX_ (Fig. 8) either. The expression of KCND3 (I_to_) was increased (Fig. 4E), consistent with the increased currents (Figure S5A,B). The expression of KNCH2 (I_Kr_), CACNA1C (I_Ca-L_) and ABCC8 (K_ATP_ , beta-subunit) was also increased (Fig. 4C, I and K) but not consistent with the current measurements (Figure S5–S7). In addition, the expressions of KCNQ1 (I_Ks_), KCNK3 (TASK-1) and KCNJ11 (KATP channel, alpha subunit) were not significantly influenced by LPS (Fig. 4F, G, H), same as the current measurements (Figure S6 and S7). Given the inconsistency in patch-clamp and qPCR measurements, further studies are needed to clarify the mechanisms underlying observed changes in ion channel currents. Nevertheless, the patch-clamp study, a widely accepted and used technique for functional analysis of ion channels, demonstrated in this study clearly that hiPSC-CMs exhibit ion dysfunctions when irritated by LPS.

In summary, by using the established hiPSC-CM platform we assessed intensively the LPS-induced responses including changes in inflammatory cytokines, cell-injury, apoptosis, necrosis, and electrophysiological properties. The results demonstrated that hiPSC-CMs possess the necessary molecular basis of the inflammatory reaction system and displayed inflammatory responses with electrical dysfunctions when challenged by LPS. This indicates that hiPSC-CMs can model some bacterium-induced inflammatory heart diseases, at least in certain aspects and might be a good alternative for mechanistic and therapeutic studies on some inflammatory cardiac diseases.

## Study limitations

Some limitations should be considered in extrapolating the data from the current study. hiPSC-CMs from only one healthy donor were used for this study. hiPSC-CMs possess similarities but also distinct differences in their physiological properties when compared to adult human cardiomyocytes. Owing to the limited availability of cells, native human cardiomyocytes were not used for comparison in this study. Therefore we cannot rule out the differences among individuals and between hiPSC-CMs and native human cardiomyocytes regarding the responses to LPS. In addition, the reasons for some discrepancies in ion channel studies were not clarified. Some mRNA expressions are not consistent with the current measurements. Therefore the mechanisms underlying the observed changes of ion channel currents need to be addressed in further studies.

## Material and Methods

### Ethics statement

The skin biopsy from a healthy donor was obtained with written informed consent. The study was approved by the Ethics Committee of Medical Faculty Mannheim, University of Heidelberg (approval number:2009-350N-MA) and conducted in accordance with the Helsinki Declaration of 1975, as revised in 1983.

### Generation of human iPS cells

The human iPS cells (hiPSCs) were generated from primary human fibroblasts derived from skin biopsies of a female healthy donor using lentiviral particles carrying the transactivator tTA and an inducible polycistronic cassette containing the reprogramming factors OCT4, SOX2, KLF4 and c-MYC as previously described^42, 43^. Generated hiPSCs were cultured under feeder free conditions. To investigate pluripotency, hiPSCs were subjected to a teratoma-formation assay^42^.

### Generation of hiPSC-CMs

Frozen aliquots of hiPSCs were thawed and cultured without feeder cells and differentiated into hiPSC-CMs as described with some modifications^44^. Culture dishes and wells were coated with Matrigel (Corning). Culture medium of hiPSCs was TeSR-E8 (Stemcell Technologies) and for hiPSC-CMs we used RPMI 1640 Glutamax (Life Technologies) containing sodium pyruvate, Penicillin/Streptomycin, B27 (Life Technologies) and ascorbic acid (Sigma Aldrich). Adding of CHIR99021 (Stemgent), BMP-4 (R&D Systems), Activin A (R&D Systems), FGF-2 (MiltenyiBiotec) and IWP-4 (Stemgent) at different time points induced the cells to differentiate into hiPSC-CMs during 3 weeks. During the third week a lactate (Sigma, Germany) containing RPMI-medium without glucose and glutamine (WKS, Germany) was added to select for cardiomyocyte cells. At 30 to 60 days of culture with basic culture medium, cardiomyocytes were dissociated from 24 well plates and plated as single cells on matrigel-coated 3.5 cm petri dishes for patch-clamp measurements. The cells were incubated with 300 µl (150 U) collagenase CLS I (Worthington, Germany) for 40 min at 37 °C, washed with PBS and incubated with 0.05% Trypsin-EDTA (Life Technologies) for 4 min at 37 °C. After adding of RPMI medium containing 10% FCS, cells were centrifuged at 250 × g for 2 min at room temperature, the supernatant discarded and the cells resuspended with basic culture medium. The cells were plated on the 3.5 cm petri dishes at a density of 2–4 × 10^4^ cells/dish for subsequent patch-clamp experiments.

### Polymerase-Chain-Reaction Assays

For quantitative evaluation of the steady-state mRNA expression in hiPSC-CM cultures, total RNA was prepared using the RNeasy mini kit (Qiagen, Hilden, Germany), including DNAse treatment. The RNA was reverse transcribed to cDNA with oligo (dT)_15_ primers using AMV reverse transcriptase according to standard protocols (Roche Applied Science, Germany, AMV Cat. No.11495062001). The cDNA was amplified by qPCR on a Stratagene MX 3005 P real time cycler (Stratagene, USA) using a PCR-mix with hot start Taq DNA polymerase and SYBR-Green (SibirRox Hot Mastermix, Bioron, Germany, Cat No. 119405) in the presence of sense and antisense primers (400 nM each). The sense- and antisense-primers for all human genes were supplied as RT² qPCR Primer Assays from Qiagen, Germany (see supplementary material Table 1, list of genes, RefSeq numbers and primers). The PCR condition consisted of 95 °C for 5 min (denaturation of DNA and activation of Taq polymerase), followed by 40 cycles of 95 °C for 15 sec and 60 °C for 1 min (annealing and extension), followed by melting-curve analysis to verify the correctness of the amplicon. Relative quantification of mRNA expression was calculated as follows: The expression of the mRNA of the gene of interest relative to the housekeeping gene GAPDH in samples from treated or untreated (Control) cells was calculated by the ΔΔCT method, based on the threshold cycle (CT), as fold change = 2^−Δ(ΔCT)^, where ΔCT = CT_gene of interest_ − CT_GAPDH_ and Δ(ΔCT) = ΔCT_treated_ −ΔCT _Control_^45^. Four equally treated wells were pooled to one biological replica to enrich the RNA-concentration. Every experiment was made with at least 2 biological replica. qPCR analysis of each biological replica was measured in duplicate (2 technical replica). The GenBank NCBI reference sequence number (RefSeq No.) and catalog number of all the investigated genes are listed in Table S1.

### Enzyme-Linked Immunosorbent Assays (ELISA)

Concentrations of human interleukin 6, sCD130 and troponin t were measured in the cell culture supernatants by ELISA (RayBiotech:ELH-IL6, ELH-sgp130 and ELH-troponin t) according to the manufacturer’s instructions. The dilution of the supernatants was 1:2.

### Flow cytometric analysis (FACS)

Cardiomyocytes were dissociated from cell culture plates as described in “Generation of hiPSC-CMs”. Afterwards cells were washed with PBS, fixed with 1% formaldehyde solution (Merck) and permeabilized with perm/wash buffer for 10 minutes (1:10 dilution, BD Bioscienes cat# 554723). Then cells were incubated with different antibodies for 30 minutes at 4 °C in the dark. Finally antibody solution was washed out with PBS. Samples were measured immediately by FACS analysis, which was performed with CANTO II (BD). Compensation-measurement was considered if necessary by using OneCompeBeads (eBioscience). All antibodies were checked for correct concentration and non-specific fluorescence signal by previously testing with titration (1:10000 to 1:10 dilution) and comparison to negative control respectively by using isotype controls. The antibodies used for FACS analysis were AlexaFluor 647-conjugated cardiac Troponin T (1:10000 dilution, BD Biosciences cat# 565744), and PerCP/Cy5.5-conjugated CD 126 antibody (1:20 dilution, BioLegend cat# 352812). Apoptosis- and necrosis- ratio of LPS-treated hiPSC-CMs were measured with the Annexin-V-APC/7-AAD Kit (BioLegend cat# 640930, 420201).

### Immunofluorescence staining

Cardiomyocytes were dissociated from cell culture plates as described in “Generation of hiPSC-CMs”. The separated cells were resuspended with serum-free medium and transferred to 4 chamber glass slides (Corning). After resting for 48 h the cells were washed with PBS. Fixation was performed using 4% paraformaldehyde (Roti-Histofix Roth cat# P087.4) for 10 minutes. Afterwards the cells were permeabilized for 10 minutes with 0,5% Triton X-100 (Sigma cat# T-9284) and blocked with PBS containing 1% bovine serum albumin (Sigma cat# A-6003). After each step they were washed with PBS. Finally cells were incubated with several antibodies at 4 °C in the dark. After one hour antibodies were washed out with PBS. Nuclear staining was performed with Vectashield mounting medium with DAPI (Linaris cat# H-1200). The antibodies used in this study were FITC-conjugated cardiac troponin T antibody (1:66 dilution, biorbyt cat# orb187249), cy5-conjugated titin antibody (1:66 dilution, BiossInc cat# bs-9861R-Cy5), Alexa Fluor 488-conjugated NfκB-p65 subunit antibody (1:40 dilution, BD biosciences cat# 558421). Pictures were taken with Leica DMRE fluorescence microscope, DFC3000G/DFC 450c camera equipped with Leica Application suite 4.4 software.

### Measurement of intracellular calcium concentration

To measure the intracellular Ca^2+^ concentration ([Ca^2+^]_i_), cells were loaded with the fluorescent Ca^2+^-indicator Fluo-3 AM. First, 1.5 ml PSS (see below) was added into a petri dish with hiPSC-CMs cultured for 2 to 4 days. The following steps were executed under consideration of the light sensitivity of the fluorescent Ca^2+^ Indicator Fluo-3. Then, 50 µg of the membrane permeable acetoxymethyl ester derivative of Fluo-3 was dissolved in 44 µl of the Pluronic F-127 stock solution (20% w/v in DMSO) to get a 1 mM Fluo-3 AM stock solution, which can be stored at −20 °C for a maximum of 1 week. Next, 15 µl of the Fluo-3 AM stock solution were added into 1.5 ml PSS resulting in a final concentration of 10 µM Fluo-3 and the dish was agitated carefully. The cells were incubated at room temperature for 10 minutes in an optically opaque box to protect from light. Thereafter, the PSS was carefully sucked out and discarded and the cells were washed with PSS for 4–5 times. Finally, the cells in PSS were kept at room temperature for about 30 minutes for de-esterification before measurements. After de-esterification the fluorescence of the cells was measured by using Cairn Optoscan (Cairn Research, UK) calcium imaging system. Fluorescence is excited by 488 nm and emitted at 520 nm. Changes in [Ca^2+^]_I_ were described by

${[C{a}^{2+}]}_{i}={k}_{d}(\frac{F}{{F}_{\max }-F})$, where *k*_*d*_ = dissociation constant of Fluo-3 (864-nmol/L), *F* = Fluo-3 fluorescence; *F*_*max*_ = Ca^2+^-saturated fluorescence obtained at the end of each experiment^46^.

### Patch-clamp

Standard patch-clamp recording techniques were used to measure the action potential (AP) and ion channel currents in the whole-cell configuration. Patch electrodes were pulled from borosilicate glass capillaries (MTW 150F; world Precision Instruments, Inc., Sarasota, FL) using a DMZ-Universal Puller (Zeitz-Instrumente Vertriebs GmbH, Martinsried, Germany) and filled with pre-filtered pipette solution (see below). Pipette resistance ranged from 1–2 MΩ. Electrode offset potentials were zero-adjusted before a Giga-seal was formed. After a Giga-seal was obtained, fast capacitance was first compensated and then the membrane under the pipette tip was disrupted by negative pressure to establish the whole-cell configuration. To determine the cell capacitance, a voltage pulse from −80 to −85 was given to record the cell capacitance transient current. Then we integrated the area under transient current curve and divide the area value by 5 mV to get the whole cell capacitance in pF. Thereafter the membrane capacitance (Cm) and series resistance (Rs) were compensated (60–80%). Liquid junction potentials were not corrected. Signals were acquired at 10 kHz and filtered at 2 kHz with the Axon 200B amplifier and Digidata 1440A digitizer hardware as well as pClamp 10.2 software (Molecular Devices, Sunnyvale, CA). Myocytes were held at different potentials and different voltage clamp protocols were used measuring different currents. Measured currents were normalized to the membrane capacitance. Current-voltage (I-V) relationships were generated by plotting the current density against voltages. For assessing the activation and inactivation of peak I_Na_, the membrane conductance or relative current (I_m_/I_max_) were plotted against voltages and then fitted with Boltzmann distribution to obtain the half maximal voltage (V0.5) of activation or inactivation. The membrane conductance (G_m_) was calculated as G_m_ = I/(E_m_ − E_rev_), where I is macroscopic current, E_m_ is the test membrane potential, and E_rev_ is the reversal potential. To measure the recovery from inactivation of sodium channels, the protocol of double-pulse with increasing intervals was used. The peak I_Na_ elicited by the second pulse was normalized to that elicited by the first pulse and plotted against the intervals between the two pulses and then fitted with mono-exponential growth to get the time constant of recovery. The TTX sensitive late I_Na_ was measured as the area under the current curve integrated from 50 to 350 ms after the beginning of the depolarization pulse. To isolate different ion channel currents, specific channel blockers were used and the blocker-sensitive currents were analyzed. To minimize the effects of run-down of recorded currents on the results of experiments, we carefully monitored the time-dependent change of currents. Recordings were started after the current reached a steady state, normally within 3 to 5 minutes.

The bath solution for AP and K^+^ channel current measurements contained (mmol/l): 130 NaCl, 5.9 KCl, 2.4 CaCl_2_, 1.2 MgCl_2_, 11 glucose, 10 HEPES, pH 7.4 (NaOH). For the I_to_ measurements, 10 µM nifedipine, 10 µM TTX and 1 µM E-4031 were added in the bath solution to block I_Ca-L_, I_Na_ and I_Kr_. For I_Kr_ and I_Ks_ measurements, 10 µM nifedipine, 10 µM TTX and 5 mM 4-AP were added. The pipette solution contains 10 mM HEPES, 126 mM KCl, 6 mM NaCl, 1.2 mM MgCl_2_, 5 mM EGTA, 11 mM glucose and 1 mM MgATP, pH 7.4 (KOH). For measuring SK channel currents, appropriate CaCl_2_ was added to get the free-Ca^2+^ concentration of 0.5 µM according to the calculation by the software MAXCHELATOR (http://web.stanford.edu/~cpatton/downloads.htm). For measuring I_KATP_ (ATP-sensitive K^+^ channel current), the ATP-free pipette solution was used.

The bath solution for peak sodium current measurements contained (mmol/l): 20 NaCl, 110 CsCl, 1.8 CaCl_2_, 1 MgCl_2_, 10 Hepes, 10 glucose, 0.001 nifedipine, pH 7.4 (CsOH). Microelectrodes were filled with (mmol/l): 10 NaCl, 135 CsCl, 2 CaCl_2_, 3 MgATP, 2 TEA-Cl, 5 EGTA, 10 HEPES, pH7.2 (CsOH).

The bath solution for late sodium current measurements contained (mmol/l): 135 NaCl, 20 CsCl, 1.8 CaCl_2_, 1 MgCl_2_, 10 Hepes, 10 glucose, 0.001 nifedipine, pH 7.4 (CsOH). Microelectrodes were filled with (mmol/l): 10 NaCl, 135 CsCl, 2 CaCl_2_, 3 MgATP, 2 TEA-Cl, 5 EGTA, 10 HEPES, pH7.2 (CsOH).

The bath solution for I_Ca-L_ recordings contained (mmol/l): 140 TEA-Cl, 5 CaCl_2_, 1 MgCl_2_, 10 3R4S-chromanol 293B, 10 Hepes, 0.01 TTX, 2 4-AP, pH 7.4 (CsOH). Microelectrodes were filled with (mmol/l): 10 NaCl, 135 CsCl, 2 CaCl_2_, 3 MgATP, 2 TEA-Cl, 5 EGTA, 10 HEPES, pH7.2 (CsOH).

The bath solution for I_NCX_ measurements contained (mmol/l): 135 NaCl, 10 CsCl, 2 CaCl_2_, 1 MgCl_2_, 10 Hepes, 10 glucose, 0.01 nifedipine, 0.1 niflumic acid, 0.05 lidocaine, 0.02 dihydroouabain, pH 7.4 (CsOH). Microelectrodes were filled with (mmol/l): 10 NaOH, 150CsOH, 2 CaCl_2_, 1 MgCl_2_, 75 aspartic acid, 5 EGTA, pH7.2 (CsOH).

### Drugs

E-4031, chromanol 293B, nifedipine, NiCl_2_, NS8593, niflunic acid, LPS (Lipopolysaccharides from E. coli, source strain ATCC 12740, serotype 0127:B8, gel filtrated, gamma irradiated, cell culture tested, Sigma L 4516), lidocaine, glybenclamide and dihydroouabain are from Sigma Aldrich, 4-AP from RBI, apamin from Alomone Labs, TTX from Carl Roth. E-4031, NiCl_2_, TTX, 4-AP, apamin, niflumic acid, glybenclamide and dihydrooubain were dissolved in H_2_O. Nifedipine, NS8593 and chromanol 293B were dissolved in DMSO, lidocaine in ethanol and LPS in RBMI medium. Stock solutions were kept at −20 °C.

### Statistics

If not otherwise indicated data are shown as mean ± SEM and were analyzed using InStat© (GraphPad, San Diego, USA) and SigmaPlot 11.0 (Systat GmbH, Germany). By analyzing the data with the Kolmogorov Smirnov test it was decided whether parametric or non-parametric tests were used for analysis. For parametric data one-way ANOVA with Dunnett’s post-test for multiple comparisons (all treated groups versus control) or test of trend were performed. For non-parametric data the Kruskal-Wallis test with Dunn’s multiple comparisons post-test was used. For repeated measurements, e.g. analysis of current-voltage relationships, parametric one-way repeated measures ANOVA with Dunnett’s multiple comparisons post-test was applied. Unpaired Student’s t-test was used for comparisons of two independent groups with normal distribution. *p* < 0.05 (two-tailed) was considered significant*, p* < 0.10 (two-tailed) was considered significant by tendency.

## Electronic supplementary material

Supplemental data
