# Supplementary material for: Lipopolysaccharides induced inflammatory responses and electrophysiological dysfunctions in human-induced pluripotent stem cell derived cardiomyocytes
Source: Sci Rep. 2017 Jun 7;7:2935. doi: 10.1038/s41598-017-03147-4 (PMC5462745; doi:10.1038/s41598-017-03147-4)
Supplement: Supplementary file 1 — Supplemental data [file 41598_2017_3147_MOESM1_ESM.pdf]

Lipopolysaccharides induced inflammatory responses and electrophysiological dysfunctions in human-induced pluripotent stem cell derived cardiomyocytes

Gökhan Yücel[1,2*], Zhihan Zhao[1,2*], Ibrahim El-Battrawy[1,2], Huan Lan[1,6], Siegfried Lang[1,2], Xin Li[1], Fanis Buljubasic[1,2], Wolfram-Hubertus Zimmermann[3,2], Lukas Cyganek[2,7], Jochen Utikal[4,2], Ursula Ravens[8], Thomas Wieland[5,2], Martin Borggrefe[1,2], Xiao-Bo Zhou[1,2,6], Ibrahim Akin[1,2]

[1]First Department of Medicine, Faculty of Medicine, University Medical Centre Mannheim (UMM), University of Heidelberg, Mannheim, Germany

[2]DZHK (German Center for Cardiovascular Research), Partner Sites, Heidelberg-Mannheim and Göttingen, Germany

[3]Institute of Pharmacology and Toxicology, University of Göttingen, Göttingen, Germany

[4]Skin Cancer Unit, German Cancer Research Center (DKFZ), Heidelberg and Department of Dermatology, Venereology and Allergology, University Medical Center Mannheim, University of Heidelberg, Mannheim, Germany;

[5]Institute of Experimental and Clinical Pharmacology and Toxicology, Medical Faculty Mannheim, University of Heidelberg, Mannheim, Germany

[6]Key Laboratory of Medical Electrophysiology of Ministry of Education, Institute of Cardiovascular Research, Southwest Medical University, Luzhou, Sichuan, China

[7] Stem Cell Unit, Heart Research Center Göttingen, Göttingen, Germany

[8] Institute of Experimental Cardiovascular Medicine, University Heart Centre Freiburg•Bad Krozingen, Freiburg, Germany

*equally contributed

Address for correspondence:

Xiao-Bo Zhou, MD

First Department of Medicine

University Medical Centre Mannheim

Theodor-Kutzer-Ufer 1-3

68167 Mannheim

Germany

E-mail: Xiaobo.zhou@medma.uni-heidelberg.de

Legends for supplemental data

Table S1. List of genes, RefSeq numbers and primers for qPCR.

Figure S1. Effects of LPS on IL-6 signaling. A, IL-6 concentration in supernatants of hiPSC-CM, which was significantly increased after LPS-treatment in different concentrations for 6h (white bar) or 48h (gray bar) (one-way ANOVA: 6h, $p<0.0001$; 48h, $p<0.0001$). **B,** Representative recordings of cardiac TNNT (Tnnt2) and CD126 from FACS-analysis of hiPSC-CM. The positive signal for cardiac Tnnt2 was detected (P2 gate). But there was no specific antibody binding leading to fluorescence signal for CD126 (P3 gate), either with or without LPS-treatment, suggesting that there was no expression of CD 126 in hiPS-CMs. **C,** sCD130 (gylcoprotein 130) concentration in supernatants of hiPSC-CM after LPS treatment in different concentrations for 6h (white bar) or 48h (gray bar). Although 6h-treatment had no influence on sCD130 concentration, 48h-treatment with LPS in high concentrations raised sCD130 concentrations (one-way ANOVA: 6h, p=0,51; 48h, p=0,019).

Figure S2. Immunostaining of hiPSC-CM for cardiac structure proteins and NFκb. Nuclear staining was induced with DAPI (blue). **A-B**, FITC-conjugated cTNNT2 antibody at day 30 after differentiation (green). **C**, FITC-conjugated cTNNT2 antibody (green) plus cy5-conjugated titin antibody (red). **D**, FITC-conjugated NFκb-p65 subunit antibody (green) after cardiomyocyte treatment with 10µg/ml LPS for 6 hours, showing the nuclear-near signal.

Figure S3. Effect of LPS on apamin-sensitive currents. Membrane currents were recorded in cells treated by either vehicle (A-C) or 1 µg/ml LPS (D-F) for 48h. **A** and **D**, Representative traces of membrane currents from -80 to +80 mV in absence of apamin. **B** and **E**, Representative traces of membrane currents from -80 to +80 mV in presence of 100 nM apamin. **C** and **F**, Representative traces of apamin-sensitive currents.

Figure S4. Effect of LPS on NS8593-sensitive currents. Membrane currents were recorded in cells treated by either vehicle (A-C) or 1 µg/ml LPS (D-F) for 48h. **A** and **D**, Representative traces of membrane currents in absence of NS8593. **B** and **E**, Representative traces of membrane currents in presence of 10 µM NS8593. **C** and **F**, Representative traces of NS8593-sensitive currents.

Figure S5. Effect of LPS on transient outward currents (I_{to}) and L-type Ca^{2+} channel currents ($I_{Ca\text{-}L}$). I_{to} and $I_{Ca\text{-}L}$ were evoked by the indicated protocol (B and D) in absence (control) and presence

of LPS. 4-aminopyridine (4-AP, 5mM) was used to isolate I_{to} from other currents. **A**, Mean values of I_{to} at +80 mV. **B**, Representative I_{to}. **C**, Mean values of I_{Ca-L} at 5 mV. **D**, Representative I_{Ca-L}, Values given are mean ± SEM. n, number of cells. *$p<0.05$

Figure S6. Effect of LPS on rapidly delayed rectifier currents (I_{Kr}) and slowly delayed rectifier currents (I_{Ks}). I_{Kr} and I_{Ks} were evoked by the indicated protocol (B) in absence (control) and presence of LPS. E-4031 (1µM) was used to isolate I_{Kr} and chromanol 293B (10µM) was used to isolate I_{Ks} from other currents. **A**, Mean values of I_{Kr} at +40 mV. **B**, Representative traces of I_{Kr} at 40 mV. **C,** Mean values of I_{Ks} at 40 mV. **D**, Representative I_{Ks} at 40 mV. Values given are mean ± SEM. n, number of cells.

Figure S7. Effect of LPS on pH- and ATP-sensitive currents (I_{KATP}). A, I-V curves of alkaline (pH-8) inhibited currents in absence (control) and presence of LPS. **B**, I-V curves of acidosis (pH-6) inhibited currents in absence (control) and presence of LPS. **C**, Mean values of the pH-sensitive currents at 40 mV. **D**, I-V curves in absence and presence of either glybenclamide or nicorandil in control cells. **E**, I-V curves in absence and presence of either glybenclamide or nicorandil in LPS-treated cells. **F**, Mean values of the currents at -70 mV. Values given are mean ± SEM. n, number of cells.

Figure S8. Effects of LPS on intracellular Ca^{2+}-concentration. A and B, Representative traces of Ca^{2+}-transients in control and LPS-treated cells. C and D, Mean values of diastolic and systolic Ca^{2+}-concentration in control and LPS-treated cells. Values given are mean ± SEM. n, number of cells.

Table S1. List of genes, RefSeq numbers and primers for qPCR.

Gene symbol	RefSeq No.	Cat. No. Primers
ABCC8 (KATP, beta-subunit SUR1)	NM_000352	PPH00038F
CACNA1C (L-type Ca2+ channel)	NM_000719	PPH01378G
CCL5	NM_002985	PPH00703B
CD-14	NM_000591	PPH05723A
GAPDH	NM_002046	PPH00150F
IL1B (IL-1 beta)	NM_000576	PPH00171C
IL6	NM_000600	PPH00560C
IL8	NM_000584	PPH00568A
IL10	NM_000572	PPH00572C
KCND3 (Ito, Kv4.3)	NM_004980	PPH06923A
KCNH2 (IKr, Kv11.1)	NM_000238	PPH01660A
KCNJ11 (K_{ATP}, alpha-subunit)	NM_000525	PPH01409B
KCNK3 (TASK-1)	NM_002246	PPH08513A
KCNN2 (SK2)	NM_021614	PPH01665A
KCNN4 (SK4)	NM_002250	PPH01418C
KCNQ1 (I_{Ks}, Kv7.1)	NM_000218	PPH01419A
LBP	NM_004139	PPH01424F
Ly96 (MD2)	NM_015364	PPH06052A
MCP-1	NM_002982	PPH00192F
NfkappaB1	NM_003998	PPH00204F
RelA	NM_021975	PPH01812B
SCN10A (Na+ channel, Nav1.8)	NM_006514	PPH15064A
SCN5A (Na+ channel, Nav1.5)	NM_000335	PPH01671F
SLC8A1 (NCX1)	NM_021097	PPH12509B
TIRAP	NM_001039661	PPH06246B
TLR4	NM_138554	PPH01795F
TNF-alpha	NM_000594	PPH00341F

RefSeq No. : GenBank NCBI Reference Sequences
Cat. No. Primers: Qiagen RT² qPCR Primer Assays

Figure S1

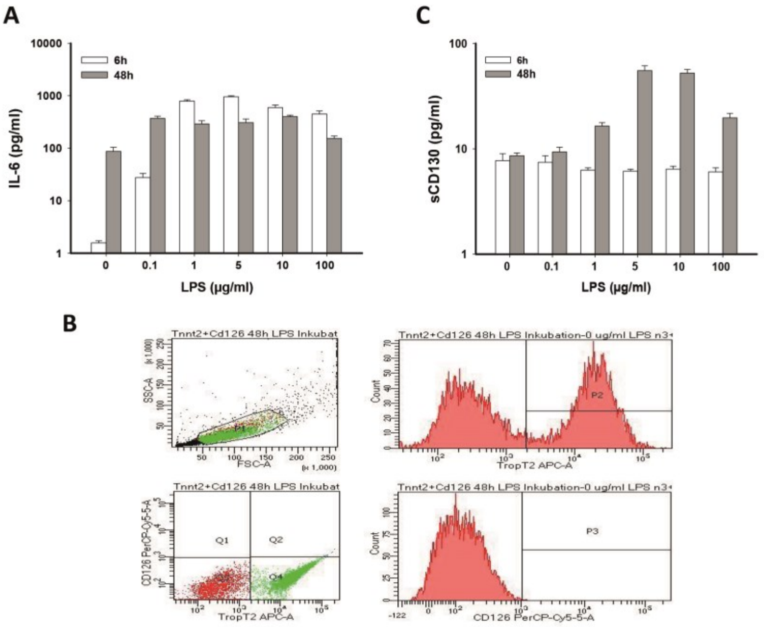

A
C
B
IL-6 (pg/ml)
sCD130 (pg/ml)
LPS (µg/ml)
6h
48h
Tnnt2+Cd126 48h LPS Inkubat
Tnnt2+Cd126 48h LPS Inkubation-0 ug/ml LPS n3
SSC-A
FSC-A
TropT2 APC-A
CD126 PerCP-Cy5-5-A
Count
P1
P2
P3
Q1
Q2
Q4

Fgure S2

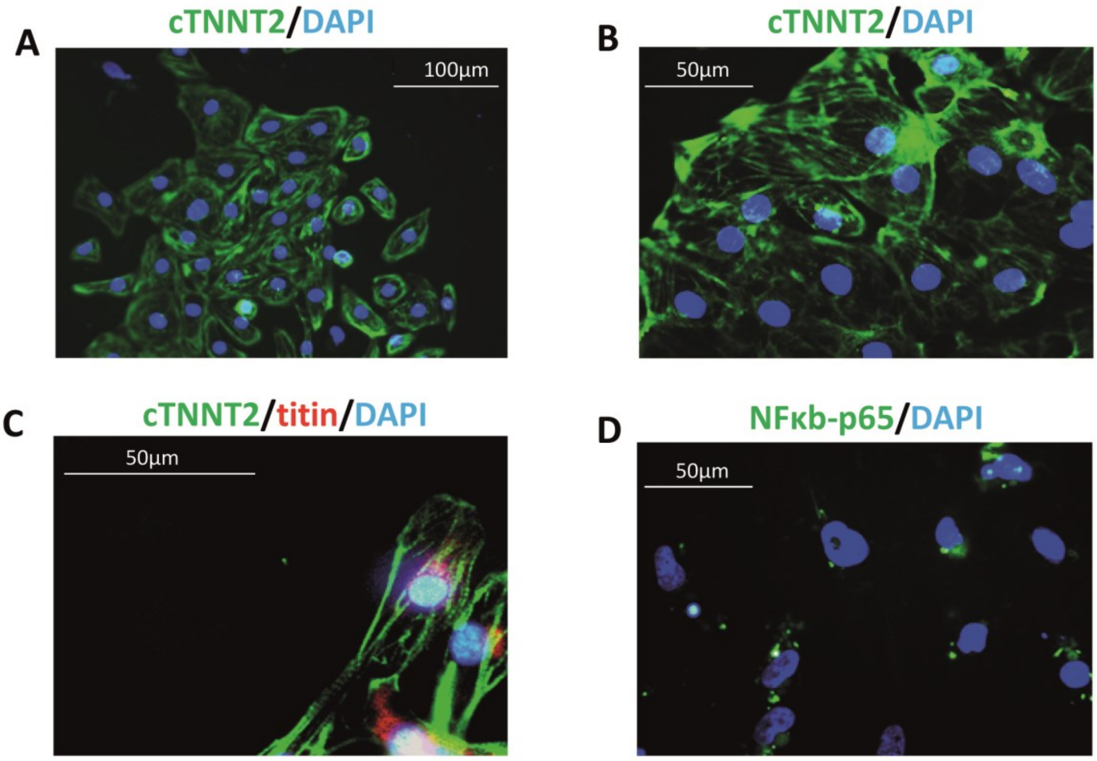
A
cTNNT2/DAPI
100µm
B
cTNNT2/DAPI
50µm
C
cTNNT2/titin/DAPI
50µm
D
NFκb-p65/DAPI
50µm

Figure S3

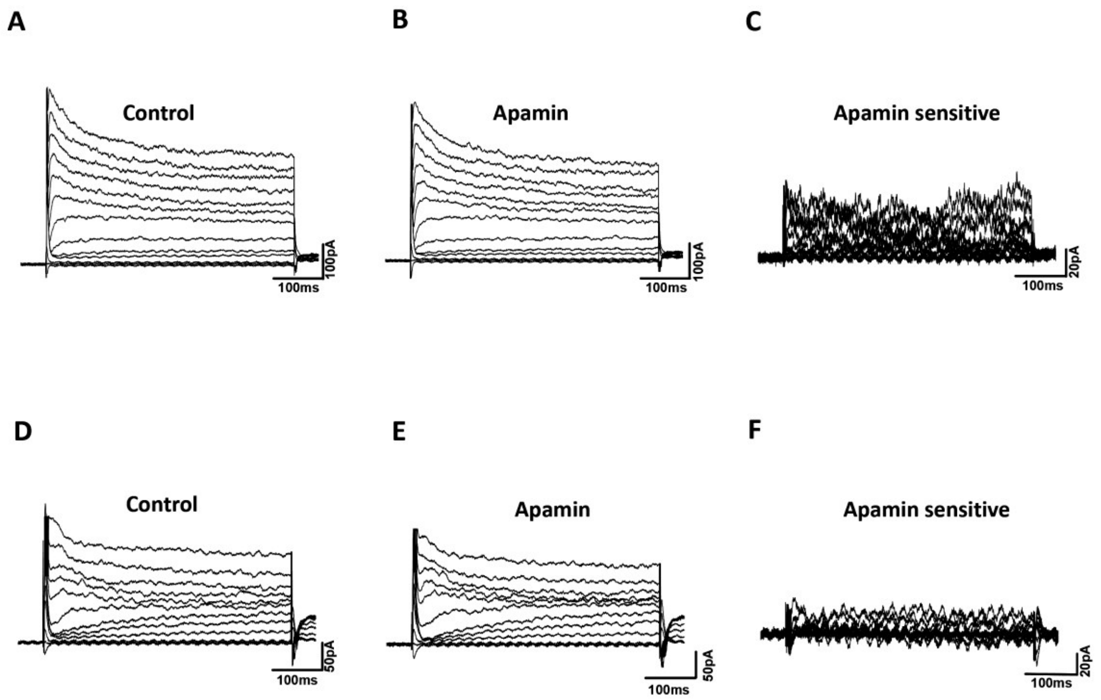
A
Control
100pA
100ms
B
Apamin
100pA
100ms
C
Apamin sensitive
20pA
100ms
D
Control
50pA
100ms
E
Apamin
50pA
100ms
F
Apamin sensitive
20pA
100ms

Figure S4

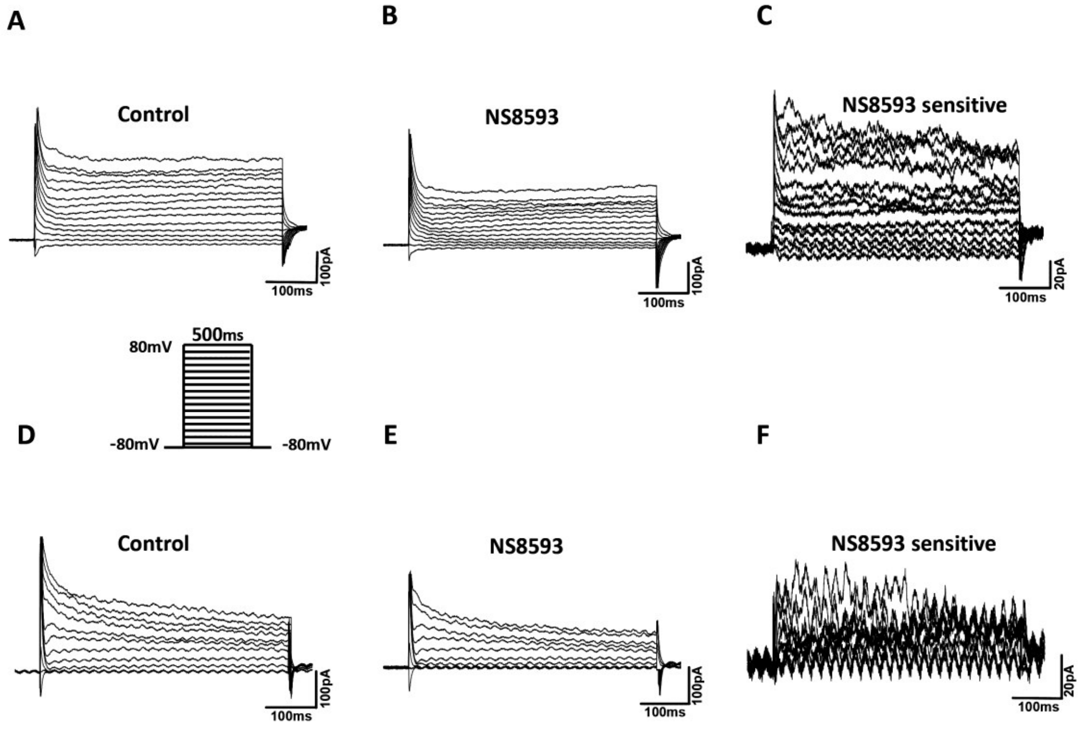
A
B
C
Control
NS8593
NS8593 sensitive
100ms
100pA
100ms
100pA
100ms
20pA
500ms
80mV
-80mV
-80mV
D
E
F
Control
NS8593
NS8593 sensitive
100ms
100pA
100ms
100pA
100ms
20pA

Figure S5

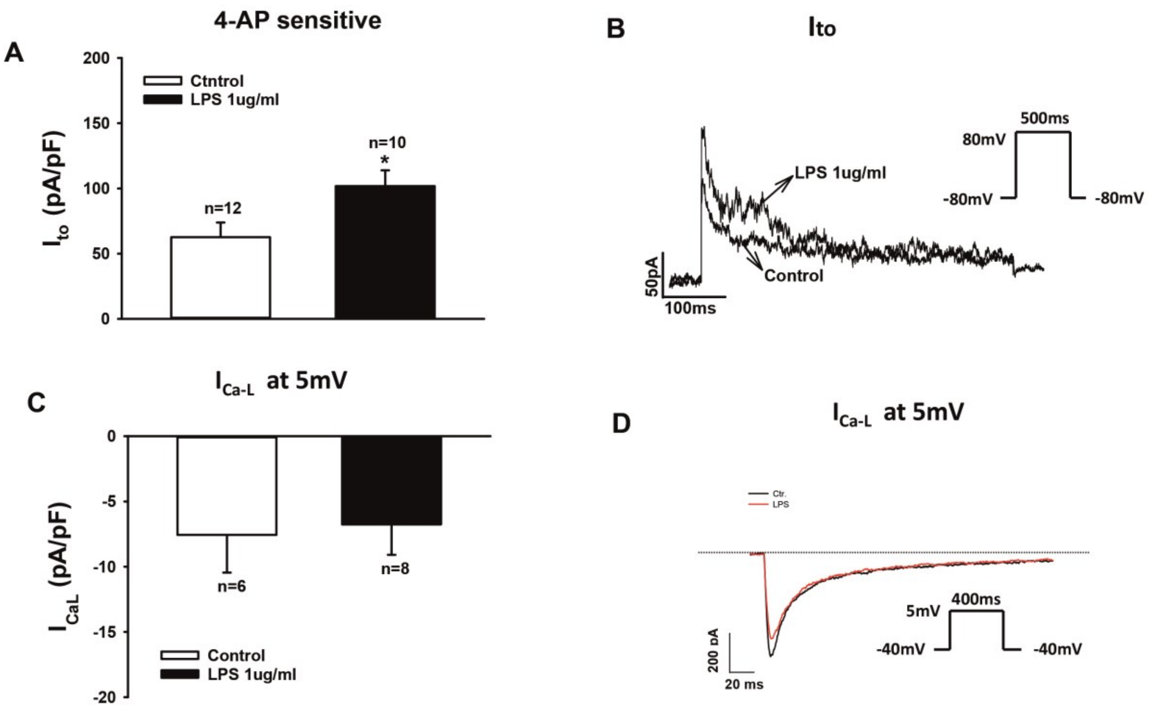

A
4-AP sensitive
Ctntrol
LPS 1ug/ml
n=12
n=10
*
I_{to} (pA/pF)
0
50
100
150
200
B
Ito
LPS 1ug/ml
Control
50pA
100ms
500ms
80mV
-80mV
-80mV
C
I_{Ca-L} at 5mV
I_{CaL} (pA/pF)
0
-5
-10
-15
-20
n=6
n=8
Control
LPS 1ug/ml
D
I_{Ca-L} at 5mV
Ctr.
LPS
400ms
5mV
-40mV
-40mV
20 ms

Figure S6

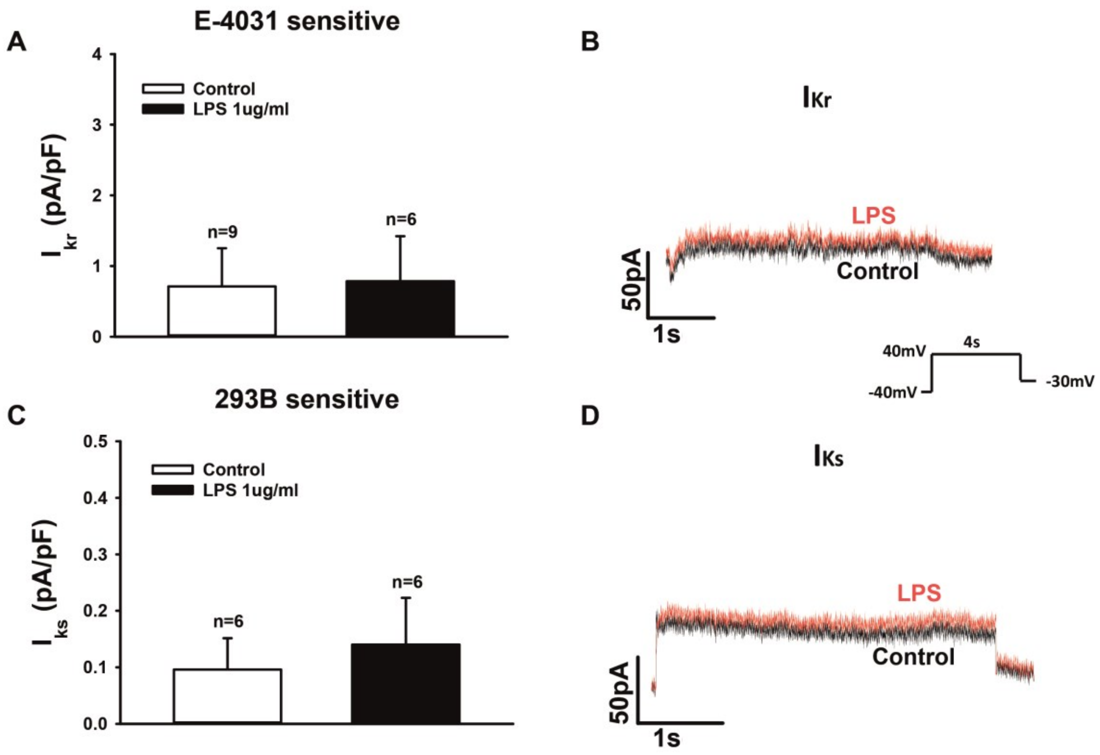
A
E-4031 sensitive
Control
LPS 1ug/ml
I kr (pA/pF)
n=9
n=6
0
1
2
3
4
B
IKr
LPS
Control
50pA
1s
40mV
4s
-40mV
-30mV
C
293B sensitive
Control
LPS 1ug/ml
I ks (pA/pF)
n=6
n=6
0.0
0.1
0.2
0.3
0.4
0.5
D
IKs
LPS
Control
50pA
1s

Figure S7

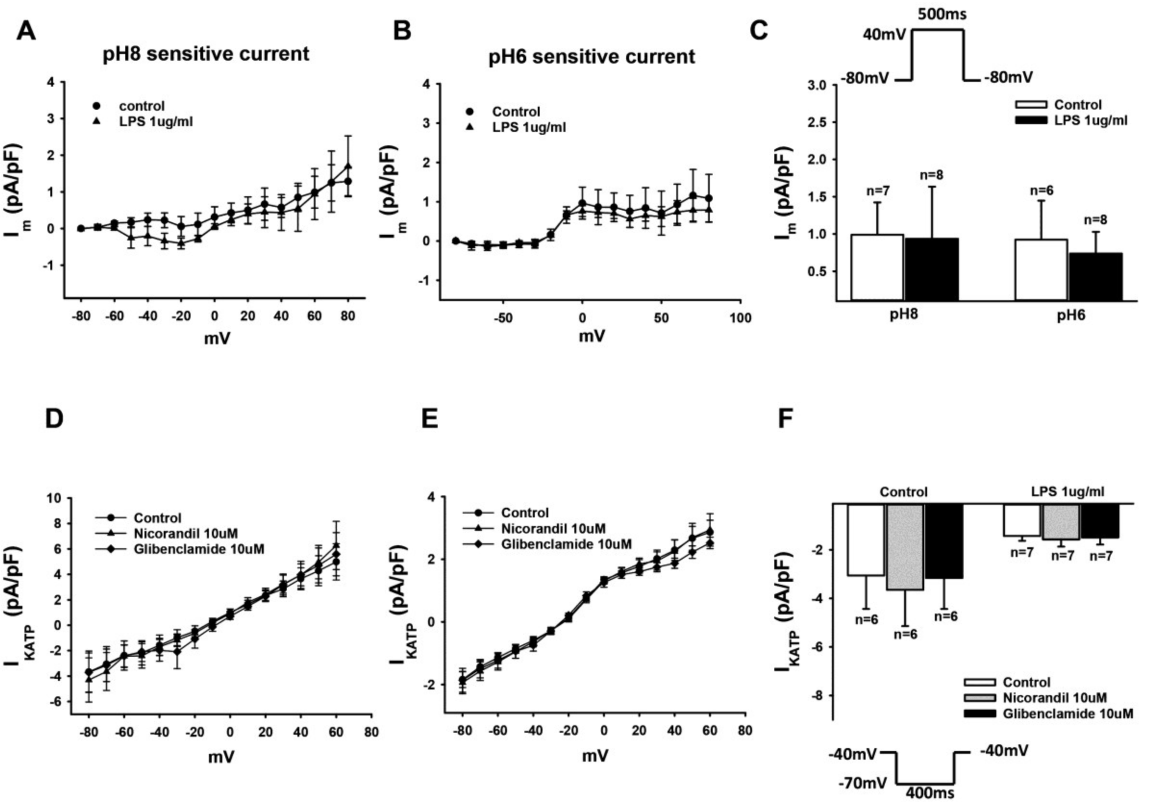

A
pH8 sensitive current
control
LPS 1ug/ml
Im (pA/pF)
mV
B
pH6 sensitive current
Control
LPS 1ug/ml
Im (pA/pF)
mV
C
500ms
40mV
-80mV
-80mV
Control
LPS 1ug/ml
Im (pA/pF)
n=7
n=8
n=6
n=8
pH8
pH6
D
Control
Nicorandil 10uM
Glibenclamide 10uM
IKATP (pA/pF)
mV
E
Control
Nicorandil 10uM
Glibenclamide 10uM
IKATP (pA/pF)
mV
F
Control
LPS 1ug/ml
n=6
n=6
n=6
n=7
n=7
n=7
IKATP (pA/pF)
Control
Nicorandil 10uM
Glibenclamide 10uM
-40mV
-70mV
400ms
-40mV

Figure S8

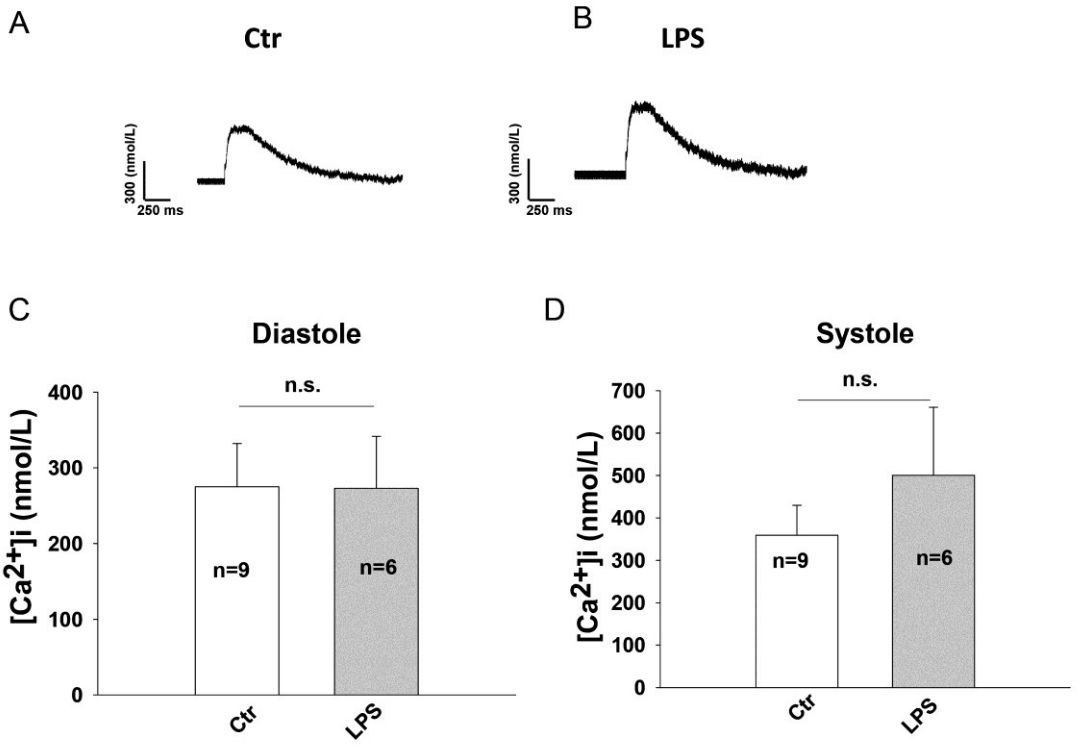
A
Ctr
300 (nmol/L)
250 ms
B
LPS
300 (nmol/L)
250 ms
C
Diastole
[Ca2+]i (nmol/L)
400
300
200
100
0
n.s.
n=9
n=6
Ctr
LPS
D
Systole
[Ca2+]i (nmol/L)
700
600
500
400
300
200
100
0
n.s.
n=9
n=6
Ctr
LPS
